# Comparative efficacy of AAC and ibuprofen versus lasmiditan, ubrogepant, and rimegepant for the acute treatment of episodic migraine in adults: a systematic review and network meta-analysis

**DOI:** 10.3389/fneur.2026.1884713

**Published:** 2026-09-15

**Authors:** Ashoke Mitra, Billy Franks, Richard Petruschke, Abhijeet Dhiman, Nitu Bansal

**Affiliations:** 1 Haleon, Warren, NJ, United States; 2 Haleon, Amersfoort, Netherlands; 3 Knowledge Centre, WNS Global Services, Gurugram, India

**Keywords:** migraine, network meta-analysis, over-the-counter, pain relief, prescription drugs

## Abstract

**Background:**

A range of pharmacologic options is available for acute migraine treatment, yet the efficacy and safety of commonly used over-the-counter (OTC) therapies relative to newer prescription agents remain unclear. Given the scarcity of direct head-to-head trials, this network meta-analysis (NMA) of randomized controlled trials (RCTs) evaluated selected OTC therapies against newer oral agents, including CGRP antagonists and the 5-HT_1_F receptor agonist.

**Methods:**

A comprehensive literature search was conducted in PubMed, Embase, and CENTRAL for RCTs of acute migraine therapies published between January 1990 and July 2025. Primary outcomes were 2-h (2 h) pain relief and pain freedom; secondary outcomes included 2-h photophobia, phonophobia and nausea. Effect sizes were calculated as relative risks (RRs) with 95% confidence intervals (CIs). A frequentist NMA was conducted to estimate comparative efficacy and safety across the treatment network.

**Results:**

This NMA synthesized evidence from 25 RCTs, including 5 different interventions across 19 dose categories, involving a total of 22,100 participants with migraine. Compared with placebo, acetaminophen, aspirin, and caffeine (AAC) combination showed superior 2 h pain relief (RR, 1.76; 95% CI, 1.61–1.94; p-score = 0.96), followed by ibuprofen 400 mg and 200 mg. For 2 h pain freedom, AAC, ibuprofen 400 mg, and lasmiditan 200 mg showed broadly comparable favorable effects versus placebo, with similar RRs and p-scores. AAC also produced the greatest reductions in photophobia and phonophobia at 2 h (photophobia: RR, 1.99; 95% CI, 1.62–2.45; p-score = 0.97; phonophobia: RR, 1.53; 95% CI, 1.28–1.84; p-score = 0.89), while ibuprofen Liquigel 400 mg provided additional meaningful benefit. For nausea, rimegepant 75 mg demonstrated the strongest effect (RR, 1.15; 95% CI, 1.08–1.22; p-score = 0.85), followed by ibuprofen Liquigel 400 mg and AAC.

**Conclusion:**

In this NMA, OTC therapies showed meaningful efficacy and favorable safety for acute episodic migraine versus placebo, with ibuprofen and AAC demonstrating consistent benefits across 2 h pain and secondary outcomes. These findings support their use as effective, accessible options alongside newer oral agents. Larger trials and patient-level analyses are needed to refine efficacy, optimize dosing, and personalize migraine management.

**Systematic review registration:**

https://www.crd.york.ac.uk/PROSPERO/view/CRD420261325571, Identifier CRD420261325571.

## 1 Introduction

Migraine is a neurologic disorder characterized by recurrent episodes of moderate to severe headache that are typically unilateral, exacerbated by physical activity, and often accompanied by phonophobia, photophobia, nausea, and vomiting (1). From 1990 to 2021, the global prevalence and incidence of migraine increased significantly by 58.15 and 42.06%, respectively, affecting over a billion people globally and the predictive analyses indicate that this trend will continue through 2050 (2). In the United States (US), the prevalence of migraine has remained stable over the past 30 years ranging from 11.7 to 14.7% overall (17.1 to 19.2% in women, and 5.6 to 7.2% in men), however migraine-related disability has increased from 22.0% in 2005 to 42.4% in 2018 (3). Reflecting on its heterogeneous nature, migraine presents predominantly as episodic migraine and less commonly as chronic migraine, which differs in attack frequency and associated burden (3). Episodic migraine, defined as headache occurring fewer than 15 days per month, represents the most prevalent migraine subtype encountered in clinical practice (4). Although attacks are intermittent, episodic migraine is associated with significant impairment in quality of life, reduced work productivity, and psychosocial distress. Consequently, the goals of acute treatment extend beyond fast and effective pain relief to include rapid and sustained restoration of function, resolution of associated symptoms, minimization of rescue medication use, and acceptable tolerability and safety (5).

A wide range of U. S. Food and Drug Administration (FDA) approved pharmacological options are available for the acute treatment of migraine in the US (6, 7). These include commonly used over the counter (OTC) agents such as nonsteroidal anti-inflammatory drugs (NSAIDs), and the fixed-dose combinations of acetaminophen, aspirin, and caffeine (AAC), as well as prescription therapies including triptans, ergots, opioids, and combination analgesics. Newly approved oral prescription agents include calcitonin gene-related peptide (CGRP) receptor antagonists (ubrogepant, rimegepant) and the selective 5-HT_1_F receptor agonist (lasmiditan), which serves as alternative treatment options (8). A recent systematic review and network meta-analysis (NMA) demonstrated that lasmiditan, rimegepant, and ubrogepant were associated with significantly higher rates of 2-h pain freedom and pain relief compared with placebo (9). Despite the availability of multiple effective therapies, the relative efficacy and safety of commonly used OTC treatments compared with newer prescription agents remain incompletely characterized. Direct head-to-head randomized trials across these drug classes are scarce, limiting robust comparative assessment and complicating evidence-based treatment selection in routine clinical practice particularly given substantial differences in accessibility, cost, and tolerability.

NMA provides a methodological framework to address these gaps by integrating direct and indirect evidence across randomized trials, enabling estimation of relative treatment effects among multiple interventions within a single analytical model (10). To our knowledge, there has been no synthesis of the evidence of the efficacy and safety of OTC treatments alongside newer oral prescription agents across both US Food and Drug Administration (FDA)-approved and non-approved dose levels for the acute treatment of episodic migraine. Therefore, we conducted a systematic review and NMA of randomized controlled trials (RCTs) to evaluate and rank the comparative efficacy of selected OTC therapies specifically the AAC combination and ibuprofen relative to newer oral prescription agents, including the CGRP antagonists (ubrogepant and rimegepant) and the 5-HT_1_F receptor agonist (lasmiditan), focusing on clinically relevant outcomes in episodic migraine.

## 2 Methods

This NMA followed the recommendations of the Preferred Reporting Items for Systematic Reviews and Meta-Analyses (PRISMA) extension statement for NMA (11). The protocol was registered in the Prospective Register of Systematic Reviews (PROSPERO CRD420261325571). A frequentist approach was adopted to synthesize direct and indirect evidence across the treatment network. The fixed-effect model was used as the primary analysis because the network included several dose-specific treatment nodes with limited direct evidence. To evaluate the influence of between-study heterogeneity, random-effects models were additionally conducted as sensitivity analyses for the efficacy outcomes. Study identification and selection were documented using a PRISMA flow diagram (Figure 1), with the completed PRISMA checklist for this meta-analysis provided in Supplementary Table S1.

**Figure 1:**
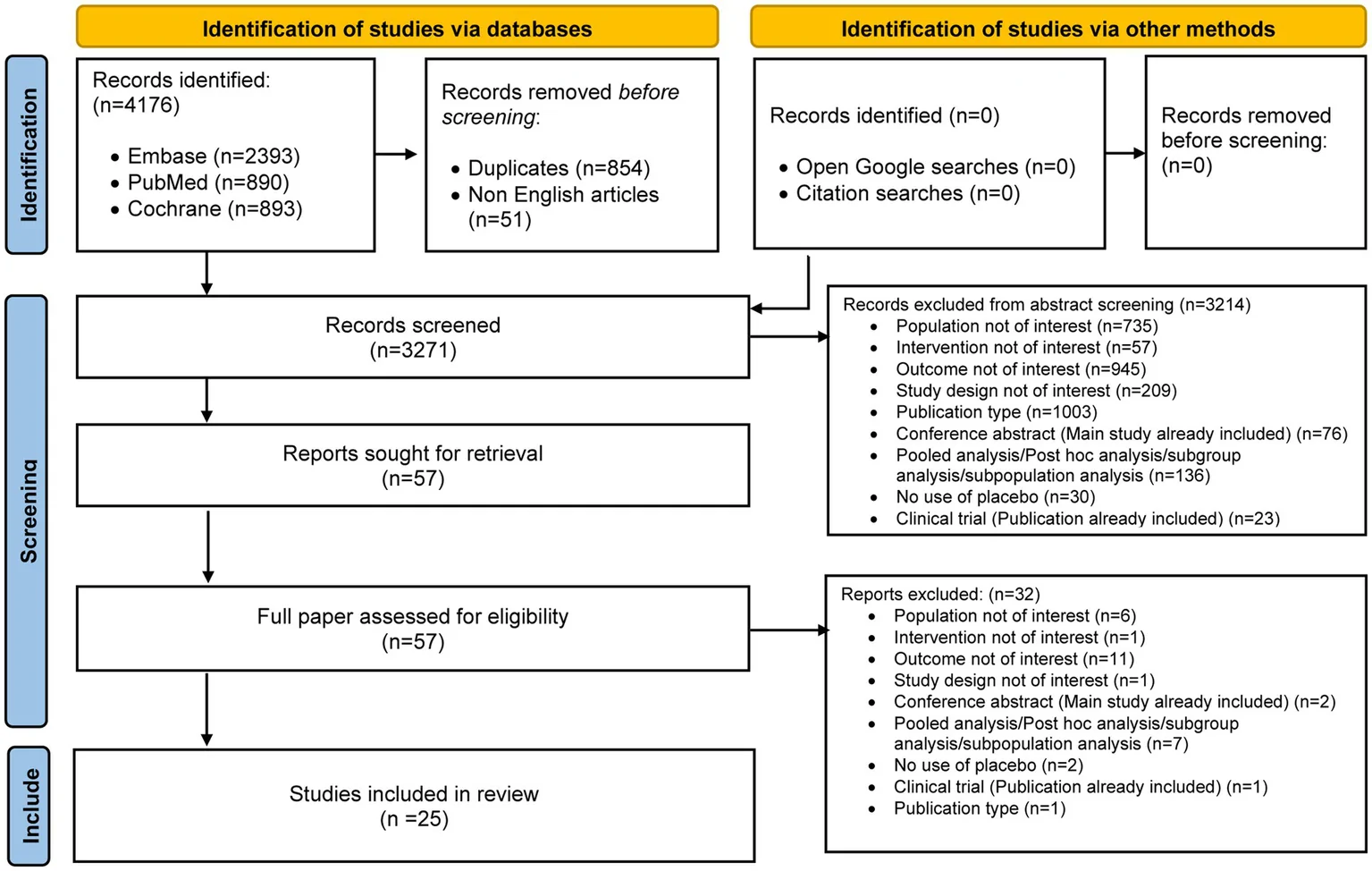
PRISMA flow chart.

### 2.1 Literature searches

A comprehensive electronic search of PubMed, Embase, and the Cochrane Central Register of Controlled Trials (CENTRAL) was conducted to identify RCTs that evaluated the efficacy and safety of approved migraine medications (including OTC and new oral prescription drugs), published in English between January 1990 and July 2025. Additionally, gray literature was searched using Google Scholar. The search strings for electronic databases consisted of combinations of keywords and Medical Subject Headings (MeSH) terms such as “ibuprofen,” “acetaminophen-aspirin-caffeine,” “lasmiditan,” “ubrogepant,” “rimegepant,” “episodic migraine,” “adult,” “headache” and “randomized controlled trial”. The complete details of the electronic search strategy and any limits applied are provided in Supplementary Table S2.

### 2.2 Study selection

Eligible studies were RCTs evaluating OTC drugs and newer oral prescription drugs for the acute treatment of migraine. Study eligibility was defined according to the PICO (Population, Intervention, Comparator, Outcome) framework as follows: (1) Participants were adult subjects with episodic migraine, defined as headache occurring on fewer than 15 days per month, diagnosed according to the International Classification of Headache Disorders (ICHD) or International Headache Society (IHS) criteria applicable at the time of each study; (2) interventions comprised of oral OTC agents, including the acetaminophen–aspirin–caffeine (AAC) combination (acetaminophen 250 mg/500 mg, aspirin 250 mg/500 mg, caffeine 65 mg/130 mg per dose) and, ibuprofen (200 mg/400 mg/600 mg), as well as newer oral prescription drugs, including lasmiditan (50 mg, 100 mg, 200 mg or 400 mg), ubrogepant (1 mg, 10 mg, 25 mg, 50 mg, or 100 mg), and rimegepant (10 mg, 25 mg, 75 mg, 150 mg, 300 mg or 600 mg), (3) compared intervention to placebo; and (4) outcomes as efficacy endpoints assessed at 2 h post-dose. Both FDA-approved and non-approved doses were included, as RCTs reported and evaluated both categories. FDA-approved doses were rimegepant 75 mg; lasmiditan 50 mg, 100 mg, and 200 mg; ubrogepant 50 mg and 100 mg; and AAC (250 mg/500 mg, 250 mg/500 mg, 65 mg/130 mg); ibuprofen 200 mg and 400 mg. All interventions included in the analysis were tablet formulations; only the ibuprofen Liquigel (200 mg/400 mg/600 mg) doses were non-tablet formulations. The primary efficacy outcomes were pain relief and pain freedom at 2 h post-dose. Pain relief was defined as an improvement in headache severity from severe or moderate to mild or no pain without the use of rescue medication, while pain freedom was defined as complete resolution of headache pain at 2 h post-dose. Across the included trials, 2-h efficacy outcomes were assessed before the use of rescue medication or were defined as outcomes achieved without rescue medication before the 2-h assessment. Secondary outcomes included reduction or absence or freedom in associated migraine symptoms (photophobia, phonophobia, and nausea) at 2 h, as defined in the original studies and the incidence of adverse events (AEs). We excluded non-RCTs, studies with incomplete or non-extractable outcomes data, conference abstracts, studies evaluating medications outside the predefined drug classes including triptans, trials involving drug combinations other than AAC or preventive migraine therapies, studies focused on prodromal symptoms, chronic migraine, tension-type headache, or nonspecific headache, and articles not published in English.

### 2.3 Study screening

Two reviewers (AD, AM) independently screened the titles and abstracts of the search results to determine whether the studies were likely to meet the inclusion criteria. Full-text articles that passed the title/abstract screening process were subsequently assessed independently by the two reviewers (AD, NB). Disagreements and uncertainties about inclusion were discussed and resolved by third reviewer (RP) if consensus could not be reached by the two reviewers.

### 2.4 Data extraction

Two reviewers (AD and NB) independently extracted data using a predesigned excel spreadsheet, capturing study characteristics (title, first author, publication year, country, treatment duration), participant details (study setting, sample size, baseline demographics), intervention details (drug name and dose), and primary outcomes assessed at 2 h post-dose (pain relief, pain freedom), and associated symptoms outcomes (photophobia, phonophobia, and nausea). Any disagreement was resolved by discussion or, when necessary, consultation with a third reviewer (RP, AM and BF).

### 2.5 Risk of bias assessment

Two reviewers (AD and NB) independently assessed the methodological quality of each RCT using the Cochrane Risk of Bias 2 (ROB 2) tool (12), applying the appropriate version for parallel-group and crossover designs. The ROB 2 tool systematically evaluates risk of bias in RCTs by assessing aspects such as randomization, adherence to intended interventions, completeness of outcome data, accuracy of outcome measurement, and selective outcome reporting (12, 13). Each domain was rated as low, some concerns and high risk.

### 2.6 Statistical analysis

We conducted NMA within a frequentist framework to synthesize direct and indirect evidence across interventions for migraine. The primary analysis used a fixed-effect model. Random-effects analyses were conducted as sensitivity analyses to evaluate the robustness of comparative estimates in the presence of between-study heterogeneity. Fixed-effect and random-effects results were compared in terms of effect direction, 95% confidence intervals, and treatment rankings. All efficacy outcomes were dichotomous, and effect sizes were calculated as risk ratios (RRs) with 95% confidence intervals (CIs). The statistical analyses were performed using the “netmeta” package in R Studio, following the intention-to-treat (ITT) approach. Direct evidence was synthesized through pairwise meta-analyses, whereas indirect evidence was estimated via the network using a common comparator. A network plot was generated to visualize the evidence structure; node size represents individual drugs at different doses, and line thickness indicates the number of studies making that comparison. All treatments were ranked based on primary and secondary outcomes with p-scores, a frequentist analog to the Surface Under the Cumulative Ranking curve (SUCRA) with a similar interpretation (14). A higher p-scores indicate a greater probability that treatment is among the most effective, with values approaching 1 suggesting the best-ranked option and values near 0 indicating the least-ranked option (14, 15). However, p-scores were interpreted as descriptive ranking measures and were considered alongside the magnitude of treatment-effect estimates, 95% CIs, consistency across outcomes, safety, and clinical considerations. Small differences in p-scores were not interpreted as evidence of clinically meaningful superiority. Network inconsistency was assessed using both global and local approaches. For the global assessment, the design-by-treatment interaction fixed-effects model provided a global test (16), and consistency was evaluated using the Q statistic and the I^2^ index (17), and the back-calculation method to distinguish direct from indirect evidence (SIDE) approach examined local inconsistencies. To examine potential small-study effects, comparison-adjusted funnel plots were utilized for outcomes that included ≥10 studies (18).

## 3 Results

### 3.1 Search results

A total of 4,176 records were identified through the literature search (Figure 1). After removal of 854 duplicates and 51 non-English articles, 3,271 records were screened by title and abstract, of which 3,214 were excluded. Full-text review was conducted for 57 articles, 32 were excluded for not meeting the inclusion criteria. Finally, 25 studies met the eligibility requirements and were included in this NMA (19–43).

### 3.2 Characteristic of included studies

All included studies were placebo-controlled RCTs published between 1997 and July 2025. Among these, 24 were parallel-group designs and one was a crossover design, collectively randomizing 26,006 participants, with 22,100 contributing to the ITT population. The study population was predominantly female (19,231 women vs. 3,588 men). Thirteen trials were conducted in the United States; three in India; two in Japan; and one in Italy, six trials recruited participants across multiple countries. Migraine diagnosis was based on IHS criteria in 16 studies and the ICHD in eight studies; in one trial, the diagnostic criteria used were not reported. Participants were mainly adults, with mean ages in the late 30s to early 40s, and a reported age range spanning approximately 30–45 years across studies. Risk-of-bias assessment indicated that 15 studies were at low risk, four were at high risk, and six had some concerns (Supplementary Figures S1a,b, S2). Detailed characteristics of the included studies, including design, diagnostic criteria, and demographic information, are summarized in Table 1. While the network included both FDA-approved and non-approved dose levels, our results were restricted to approved doses, and efficacy observed at certain non-approved doses was not incorporated into the primary interpretation.

**Table 1:** Characteristics of the included studies.

SL. No.	Author, Year	Study design	Patients randomized	ITT	Geography	Gender (F/M)	Diagnostic criteria	Migraine episodes/attacks per month	Interventions	Age*	Outcome analyzed
1	Goldstein et al., 2006 (20)	RCT, PC	1714	1,555	US	1249/306	IHS	1 × every 2 months, max 6×/month	Acet_250 mg, Aspi_250 mg, and Caff_65 mg (AAC)	38.3	1, 2
									Ibuprofen 200 mg	38.4	
									Placebo	38.3	
2	Goldstein et al., 2005 (21)	RCT, PC,	188	170	US	138/32	IHS	1 to 8	Acet_500 mg, Aspi_500 mg, and Caff_130 mg (AAC)	38.1* (Combined)	1
									Placebo		
3	Lipton et al., 1998 (22)	RCT, PC	1,357	1,247	US	964/256	IHS	1 × every 2 months, max 6×/month	Acet_250 mg, Aspi_250 mg, Caff_65 mg (AAC)	37.0 (Pooled)*	1, 2, 3, 4, 5
									Placebo	36.4	
4	Lipton et al., 2019 (23)	RCT, PC	1,686	1,465	US	1317/148	ICHD-III	2 to 8	Ubrogepant 50 mg	41.2 (12.5)	1, 2, 3, 4, 5
									Ubrogepant 25 mg	41.6 (12.4)	
									Placebo	41.7 (12.1)	
5	Voss et al., 2016 (24)	RCT, PC	834	640	US	559/81	ICHD-II	2 to 8	Ubrogepant 1 mg	39.6 (10.7)	1, 2, 3, 4, 5
									Ubrogepant 10 mg	41.1 (10.9)	
									Ubrogepant 25 mg	41.4 (11.5)	
									Ubrogepant 50 mg	40.7 (12.3)	
									Ubrogepant 100 mg	41.9 (11.0)	
									Placebo	40.5 (11.7)	
6	Dodick et al., 2019 (25)	RCT, PC	1,672	1,436	US	1266/170	ICHD-III	2 to 8	Ubrogepant 50 mg	40.1 (11.7)	1, 2, 3, 4, 5
									Ubrogepant 100 mg	40.6 (12.0)	
									Placebo	40.9 (11.7)	
7	Yu et al., 2023 (26)	RCT, PC	1,431	1,340	China and South Korea	1088/252	ICHD-III	2 to 8	Rimegepant 75 mg	37.0 (30.0–45.0) *	1, 2
									Placebo	36.0 (30.0–44·0) *	
8	Croop et al., 2019 (27)	RCT, PC	1,466	1,351	US	1147/204	ICHD-III	2 to 8	Rimegepant 75 mg	40.3 (12.1)	1, 2, 3, 4, 5
									Placebo	40.0 (11.9)	
9	Lipton et al., 2019 (28)	RCT, PC	1,186	1,072	US	951/121	ICHD-III	2 to 8	Rimegepant 75 mg	40.2 (11.9)	1, 2, 3, 4, 5
									Placebo	40.9 (12.1)	
10	Marcus et al., 2014 (29)	RCT, PC	885	776	US	645/131	NR	2 to 7	Rimegepant 10 mg	41.1 (10.4)	1, 2, 3, 4, 5
									Rimegepant 25 mg	36.5 (11.9)	
									Rimegepant 75 mg	38.5 (11.9)	
									Rimegepant 150 mg	39.2 (11.3)	
									Rimegepant 300 mg	41.9 (11.5)	
									Rimegepant 600 mg	39.3 (13.0)	
									Placebo	37.9 (11.4)	
11	Lipton et al., 2024 (30)	RCT, PC	1,162	1,084	US	927/157	ICHD-III	2 to 8	Rimegepant 75 mg	41.9 (12.3)	1, 2, 3, 4, 5
									Placebo	41.3 (12.1)	
12	Yadav et al., 2016 (19)	RCT, PC	216	143	India	96/47	IHS	<8	Ibuprofen 400 mg	33.6 (11.4)	1, 2
									Placebo	33.6 (10.3)	
13	Misra et al., 2007 (31)	RCT, PC	165	102	India	78/24	IHS	2 to 8	Ibuprofen 400 mg	30.5 (10.6)	1, 2
									Placebo	31.78 (9.9)	
14	Saper et al., 2005 (32)	RCT, PC	957	393	Asia, Europe, Latin America, and US	342/51	HIS	1 to 8	Ibuprofen 400 mg	41.3 (12.0)	1, 2, 3, 4, 5
									Placebo		
15	Misra et al., 2003 (33)	RCT, PC	124	82	India	NR	IHS	≤6	Ibuprofen 400 mg	32.3 (Combined)	1
									Placebo		
16	Diener et al., 2004 (34)	RCT, PC*	356	312	Germany, Italy and Spain	354/80	IHS	1 to 6	Ibuprofen 400 mg	38.4 (11.8)	1, 2, 3, 4, 5
									Placebo	38.3 (12.2)	
17	Codispoti et al., 2001 (35)	RCT, PC	721	660	US	556/104	IHS	1 × every 2 months, max 6×/month	Ibuprofen 200 mg	38.9 (11.3)	1, 3, 4, 5
									Ibuprofen 400 mg	38.0 (10.8)	
									Placebo	39.1 (11.1)	
18	Sandrini et al., 1998 (36)	RCT, PC	40	34	Italy	26/8	IHS	2 to 6	Ibuprofen 400 mg	34.4 (7.4)	1
									Placebo		
19	Kellstein et al., 2000 (37)	RCT, PC	972	729	US	550/179	IHS	One every 2 months to 6 times monthly	Ibuprofen 200 mg (liquigel)	37.3	1, 2, 3, 4, 5
									Ibuprofen 400 mg (liquigel)	36.7	
									Ibuprofen 600 mg (liquigel)	36.00	
									Placebo	36.4	
20	Ashina et al., 2020 (38)	RCT, PC	1,613	1,471	Europe, North America, and Asia	1236/235	IHS 1.1 and/or 1.2.1	3 to 8	Lasmiditan 100 mg	42 (12)	1,2
									Lasmiditan 200 mg	42 (12)	
									Placebo	41 (12)	
21	Sakai et al., 2020 (39)	RCT, PC	846	687	Japan	574/113	IHS 1.1 and/or 1.2.1	3 to 8	Lasmiditan 50 mg	44.9 (10.2)	1,2
									Lasmiditan 100 mg	45.7 (9.7)	
									Lasmiditan 200 mg	44.7 (10.4)	
									Placebo	45.2 (9.0)	
22	Goadsby et al., 2019 (40)	RCT, PC	2,869	2,583	UK, US, Germany	2174/409	IHS 1.1 and/or 1.2.1	3 to 8	Lasmiditan 200 mg	41.8 (12.4)	1, 2, 3, 4, 5
									Lasmiditan 100 mg	43.4 (12.6)	
									Lasmiditan 50 mg	42.8 (13.2)	
									Placebo	42.6 (12.9)	
23	Kuca et al., 2018 (41)	RCT, PC	2,231	1,671	US	1552/119	IHS 1.1 and/or 1.2.1	3 to 8	Lasmiditan 200 mg	41.4 (12.0)	1, 2, 3, 4, 5
									Lasmiditan 100 mg	42.2 (11.7)	
									Placebo	42.4 (12.3)	
24	Färkkilä et al., 2012 (42)	RCT, PC	512	391	5 European Countries	342/49	IHS 1.1 and/or 1.2.1	1 to 8	Lasmiditan 50 mg	40.4 (12.5)	1, 2, 3, 4, 5
									Lasmiditan 100 mg	42.0 (10.6)	
									Lasmiditan 200 mg	39.5 (10.3)	
									Lasmiditan 400 mg	38.7 (10.3)	
									Placebo	40.5 (10.3)	
25	Ikeda et al., 2025 (43)	RCT, PC	803	706	Japan	1100/312	ICHD-III	2 to 8	Rimegepant 25 mg	40.2 (10.4)	1, 2, 3, 4, 5
									Rimegepant 75 mg	40.5 (11.2)	
									Placebo	41.5 (11.3)	

RCT: Randomized controlled trial, PC: Placebo controlled, ITT: Intention to treat; F/M: Female/Male; Outcomes analyzed in the NMA. 1: Pain relief; 2: Pain freedom; 3: Photophobia; 4: Phonophobia; 5: Nausea. IHS: International Headache Society; ICHD-II: International Classification of Headache Disorders, second edition; ICHD-III: International Classification of Headache Disorders Criteria, 3rd edition; * Study design indicate cross over studies others are parallel; Acet: Acetaminophen, Aspi: Aspirin and Caff: caffeine (AAC); 1 × every 2 months, max 6×/month: At least once every 2 months, but no more than 6 times monthly; * Age indicate instead of mean (SD) range or combined or pooled mean is given in those studies; NR: Not reported. FDA approved doses include: AAC, Ibuprofen 200 mg and 400 mg, Lasmiditan 50 mg,100 mg and 200 mg, Rimegepant 75 mg, Ubrogepant 50 mg and 100 mg.

### 3.3 Primary efficacy outcomes

#### 3.3.1 Two-hour pain relief

The NMA on pain relief at 2 h included 25 RCTs with 5 interventions across 19 dose categories with 21,209 participants (Figure 2a). Compared with placebo the AAC combination (RR 1.76; 95% CI: 1.61–1.94), ibuprofen at doses of 400 mg (RR 1.68; 95% CI: 1.52–1.86), and 200 mg (RR 1.63; 95% CI: 1.32–2.01), demonstrated significantly superior efficacy (Figure 3a). Comparative effectiveness across all interventions is presented in the league table (Supplementary Table S3). Based on p-scores, the highest-ranked treatment was AAC (0.96) followed by ibuprofen 400 mg (0.90) and ibuprofen 200 mg (0.84) Moderate heterogeneity was observed (I^2^ = 48.5%). The comparison-adjusted funnel plot was largely symmetrical, indicating no evidence of small-study effects or publication bias (Supplementary Figure S3).

**Figure 2:**
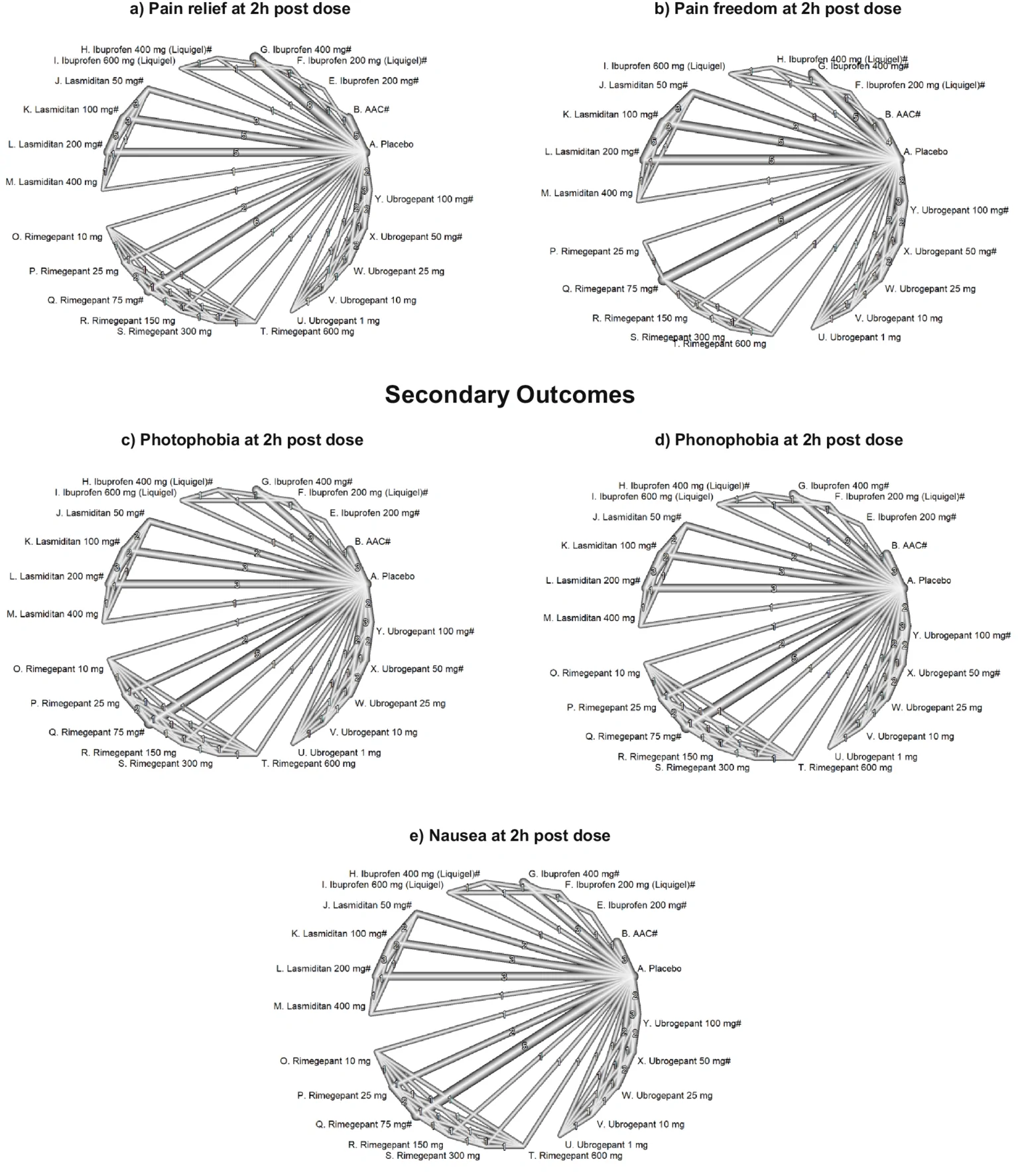
**(a–e)** Network plot for primary and secondary outcomes. The lines between nodes represent direct comparisons in various trials, and each node represents the specific treatment. The thickness of the lines is proportional to the number of trials connected to the network. “#” indicate the FDA approved doses. AAC: acetaminophen 250 mg/500 mg, aspirin 250 mg/500 mg, caffeine 65 mg/130 mg.

**Figure 3:**
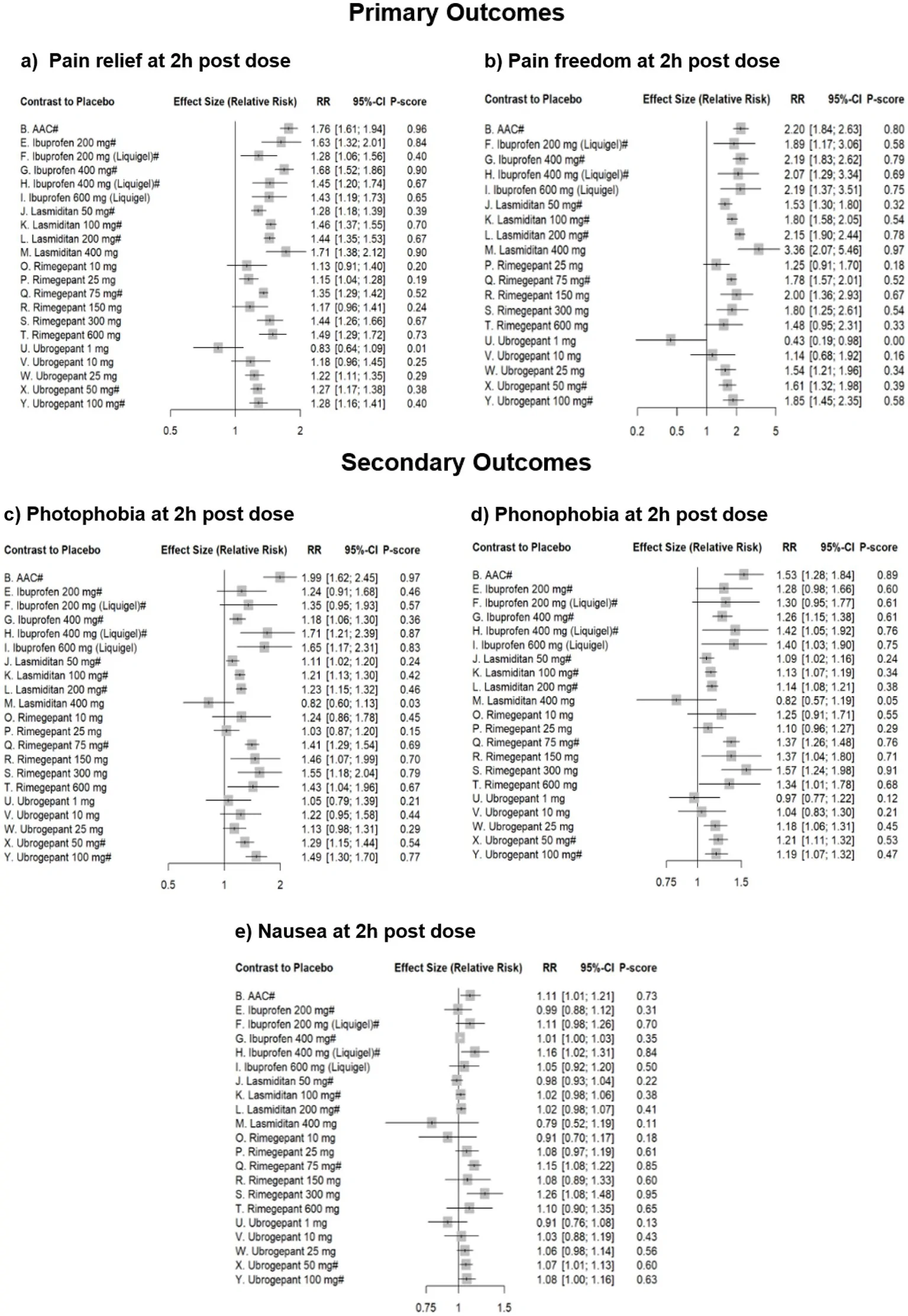
**(a–e)** Forest plot of interventions measured by pain relief, pain freedom, photophobia, phonophobia and nausea at 2 h post dose. “#” indicate the FDA approved doses. AAC: acetaminophen 250 mg/500 mg, aspirin 250 mg/500 mg, caffeine 65 mg/130 mg.

#### 3.3.2 Two-hour pain freedom

Twenty-one RCTs, including 5 interventions in 18 dose categories with 20,177 participants, contributed to the comparison of the 2-h pain freedom (Figure 2b). AAC (RR 2.20; 95% CI: 1.84–2.63), ibuprofen 400 mg (RR 2.19; 95% CI: 1.83–2.62), and lasmiditan 200 mg (RR 2.15; 95% CI: 1.90–2.44) showed similar favorable effects versus placebo for 2-h pain freedom (Figure 3b). Corresponding p-scores were also similar for AAC (0.80), ibuprofen 400 mg (0.79), and lasmiditan 200 mg (0.78) demonstrated comparable outcome. Comparative effectiveness across all interventions is presented in the league table (Supplementary Table S4). Moderate heterogeneity was present (I^2^ = 53.6%), and the comparison-adjusted funnel plot suggested no publication bias (Supplementary Figure S4).

### 3.4 Secondary efficacy outcome

#### 3.4.1 Reduction in photophobia at two hours

Sixteen RCTs (*n* = 15,882) covering 19 different dose categories were included in the analysis of photophobia at 2 h (Figure 2c). The AAC combination showed the most consistent benefit versus placebo (RR, 1.99; 95% CI: 1.62–2.45), with strong support from the network ranking (p-score = 0.97). Ibuprofen Liquigel 400 mg and ubrogepant 100 mg also produced robust effects (RR, 1.71; 95% CI: 1.21–2.39; and RR, 1.49; 95% CI: 1.30–2.70, respectively), and both ranked highly (p-scores 0.87 and 0.77 respectively). Rimegepant 75 mg also achieved modest but statistically significant improvements (RR, 1.41; 95% CI: 1.29–1.54; p score = 0.69) (Figure 3c). Lasmiditan at doses of 100 mg, and 200 mg also showed consistent benefit (Supplementary Table S5). Substantial heterogeneity was observed across the network (I^2^ = 67.8%).

#### 3.4.2 Reduction in phonophobia at two hours

A total of 16 RCTs with 15,882 participants and 18 treatment nodes were considered in assessing phonophobia at 2 h (Figure 2d). Compared to placebo, AAC demonstrated the greatest improvement in phonophobia at 2 h (RR, 1.53; 95% CI: 1.28–1.84; p-score = 0.89), followed by ibuprofen Liquigel 400 mg (RR, 1.42; 95% CI, 1.05–1.92; p-score = 0.76) and rimegepant 75 mg (RR, 1.37, 95% CI: 1.26–1.48; p-score = 0.76) (Figure 3d; Supplementary Table S6). The Q statistic indicated moderate heterogeneity for this outcome (I^2^ = 58%).

#### 3.4.3 Reduction in nausea at two hours

Sixteen RCTs (*n* = 15,882) with 19 treatment nodes were included in the analysis of nausea at 2 h (Figure 2e). Compared with placebo, rimegepant 75 mg was the highest-ranked treatment (RR 1.15; 95% CI: 1.08–1.22; p-score = 0.85) followed closely by ibuprofen Liquigel 400 mg (RR 1.16; 95% CI: 1.02–1.31; p-score = 0.84) and AAC (RR 1.11; 95% CI: 1.01–1.21; p-score = 0.73). In contrast, lasmiditan did not improve nausea at any approved dose in this network. No significant heterogeneity was observed for nausea (I^2^ = 29%) (Figure 3e; Supplementary Table S7).

### 3.5 Sensitivity analysis using random-effects models

Random-effects sensitivity analyses were conducted to evaluate the robustness of the fixed-effect findings across modeling assumptions. Overall, the direction of treatment effects and the relative positioning of the principal interventions were consistent between the fixed-effect and random-effects models. For 2-h pain relief, AAC remained favorable versus placebo in both models, with an RR of 1.76 in the fixed-effect analysis and 1.76 in the random-effects sensitivity analysis. Ibuprofen 400 mg also remained favorable versus placebo, with estimates of RR 1.68 and RR 1.74 in the fixed-effect and random-effects models, respectively. For 2-h pain freedom, AAC, ibuprofen 400 mg, and lasmiditan 200 mg remained among the better-performing approved treatments under the random-effects sensitivity analysis. For associated symptom outcomes, AAC remained favorable for photophobia and phonophobia, and rimegepant 75 mg remained favorable for phonophobia (Supplementary Figure S5). These findings support the consistency of the principal conclusions across modeling approaches.

### 3.6 Safety analysis

Across 25 randomized controlled trials (*N* = 25,912), a total of 4,271 treatment-emergent adverse events (TEAEs) (events/participants = 4,271/25,912) and 1,963 treatment-related adverse events (TRAEs) were reported. Among the evaluated treatments, lasmiditan 400 mg showed the most notable number of AEs (69/70), followed by lasmiditan 200 mg (995/2,002), 100 mg (897/2,040), and 50 mg (263/1,146). The most common AEs were nausea, dizziness, somnolence, paresthesia, and fatigue. Over-the-counter analgesics and gepants were associated with relatively fewer AEs across the studies. Safety outcomes are summarized in Supplementary Table S8.

## 4 Discussion

In this systematic review and network meta-analysis of 25 RCTs, we compared commonly used OTC analgesics, specifically the AAC combination and ibuprofen, with newer U. S. FDA approved oral therapies for acute episodic migraine, including lasmiditan, rimegepant, and ubrogepant. To our knowledge, this is the first NMA to directly compare OTC analgesics with newer oral prescription migraine therapies across the full spectrum of core two-hour outcomes, which increases the clinical relevance of the findings for frontline practitioners and health systems.

Across the network, AAC and ibuprofen 400 mg showed favorable effect estimates for two-hour pain relief and two-hour pain freedom, while AAC showed a consistent favorable profile across multiple efficacy outcomes, including pain relief, photophobia, and phonophobia. Although comparative safety could not be synthesized quantitatively, descriptive evidence suggested lower reported AEs frequencies for the AAC and ibuprofen 400 mg interventions. Taken together, these findings support the continued role of accessible and relatively low-cost analgesics as first-line options for many patients with episodic migraine, particularly in settings where access to newer agents is limited.

Among prescription therapies, lasmiditan 200 mg (the highest approved acute dose) had the highest probability of achieving two-hour pain freedom within this network. However, these benefits were accompanied by higher frequencies of AEs particularly dizziness and somnolence, consistent with a clinically important efficacy–tolerability trade-off. These findings are consistent with previous network meta-analyses, which identified lasmiditan 200 mg as among the most effective treatments for acute migraine but also associated it with higher degrees of dizziness, nausea, and somnolence than alternative therapies, including gepants (9, 44). Collectively, these results reinforce the importance of individualizing acute treatment based on patient priorities (e.g., rapid pain freedom) and practical considerations (e.g., the need to avoid impairment). In contrast, rimegepant at the approved acute dose (75 mg) provided consistent benefits for secondary outcomes, particularly photophobia, phonophobia, and nausea, but appeared less strong on pain-related outcomes at two hours relative to the highest-performing interventions. Ubrogepant demonstrated statistically significant efficacy compared with placebo at licensed doses (50 mg or 100 mg); however, its effect sizes were generally smaller than those of the top-ranked interventions in this network. Even modest short-term effects may be clinically meaningful when patient preference, contraindications, or prior non-response restrict the use of other agents.

The observed pattern of treatment effects is consistent with current understanding of migraine pathophysiology. Migraine involves complex, stage-specific neural and vascular processes, with trigeminovascular activation characterizing the headache phase (45, 46). Nonselective cyclooxygenase (COX) inhibition and reduced prostaglandin synthesis provide plausible mechanism for the rapid analgesic effect of ibuprofen and aspirin, while acetaminophen contributes central modulation of prostaglandin activity (47). Additionally, the acetaminophen and aspirin alone are not approved for the treatment of episodic migraine in the US. In contrast, the AAC combination may offer synergistic benefit, which is supported by RCTs and pharmacologic evidence that caffeine can potentiates analgesic response (48–50). By contrast, lasmiditan targets 5-HT_1_F receptors in central nociceptive pathways, and gepants antagonize the CGRP receptor, with each class offering mechanistic advantages for particular patient profiles. The findings support the conclusion that targeted mechanisms expand therapeutic options without necessarily producing uniformly superior short-term outcomes compared with optimized OTC combinations (46). Our results align with an earlier NMA by Xu et al., which suggested that ibuprofen represents an effective option with an advantageous tolerability profile, supporting its continued role in the acute treatment of episodic migraine (48). Dose-dependent patterns observed in this NMA were also concordant with prior randomized evidence and regulatory dose-selection decisions (23, 24, 29, 35, 43). Unapproved dose regimens of ubrogepant and rimegepant consistently demonstrate limited or inconsistent efficacy. For ubrogepant, lower doses have been associated with attenuated treatment effects and inconsistent achievement of freedom from the most bothersome symptom, whereas approved doses (50 mg and 100 mg) demonstrated more reliable and clinically meaningful efficacy, particularly with respect to sustained pain freedom (23, 24). Consequently, unapproved lower-dose regimens of ubrogepant were consistently positioned at the bottom of the efficacy hierarchy. For rimegepant, regulatory reviews and clinical studies demonstrated a relatively flat dose response between 75 mg and 300 mg, with no apparent efficacy observed at the 25 mg dose (49). In the pivotal efficacy trials, rimegepant was evaluated at the 75 mg dose, which was subsequently approved (29, 43). This pattern is mirrored by previous NMAs, which showed that efficacy was confined to the approved dose (50). In parallel, lasmiditan exhibited a dose-related increase in central nervous system (CNS) AEs, with the highest AE frequencies reported for the unapproved 400 mg regimen. The most commonly reported AEs included dizziness, somnolence, nausea, paresthesia, and fatigue, consistent with prior dose-ranging and evidence synthesis studies (9, 50). Collectively, these findings support the biological plausibility and internal consistency of the dose-related results observed in the network.

Patient-centered priorities for acute migraine treatment such as complete pain relief, rapid onset and prevention of recurrence align with current guideline-recommended goals and emphasize the need to balance efficacy with tolerability (51, 52). At a health-system level, the elevated cost of newer agents (ditans and gepants) can constrain access, particularly in resource-limited settings, and may widen inequities both across and within countries (53, 54). In this context, the comparable short-term performance of several OTC options supports their continued use as front-line therapies for episodic migraine, with newer prescription agents may be reserved for patients who do not respond to, cannot tolerate, or have contraindications to OTC treatments (55–57).

Although OTC analgesics were associated with relatively fewer reported adverse events in the included randomized trials, these safety findings should be interpreted in the context of short-term, mostly acute-use study designs. Therefore, the favorable safety profile observed for ibuprofen and the acetaminophen–aspirin–caffeine combination is most applicable to occasional, label-directed use. Repeated or frequent use of OTC analgesics may be associated with clinically important harms that were not fully captured in the included trials, including gastrointestinal toxicity and bleeding with aspirin- or NSAID-containing products, nephrotoxicity with NSAIDs in susceptible patients, and medication-overuse headache with frequent use of acute migraine medications. Thus, OTC treatments remain effective, accessible, and generally well-tolerated options for acute episodic migraine when used appropriately, but their use should be intermittent, label-directed, and individualized according to patient-specific risk factors.

Beyond clinical and practical considerations, the consistency of findings across modeling approaches strengthens confidence in the principal conclusions. This NMA provides a dose-specific comparison of OTC and newer prescription therapies for the acute treatment of episodic migraine. The primary fixed-effect analysis and the random-effects sensitivity analysis yielded broadly consistent treatment effects for the primary pain outcomes, with AAC and ibuprofen 400 mg remaining among the most favorable approved options. The random-effects sensitivity analyses also supported the overall interpretation of associated symptom outcomes. In addition, funnel plots did not indicate meaningful small-study effects, most included RCTs were of high methodological quality, and the evidence base was dominated by parallel-group designs. Moreover, the similarity of study designs, eligibility criteria, and patient characteristics across trials supports the transitivity assumption, thereby strengthening the validity of indirect comparisons in the network.

This study has some limitations, and it should be considered when interpreting these findings. The included studies were conducted across multiple geographic regions, although 13 of the 25 studies were performed in the United States. Therefore, the findings may not be fully generalizable to all healthcare settings and populations, particularly given differences in healthcare systems, treatment access, and clinical practice patterns across regions. In addition, the included studies predominantly enrolled women and younger to middle-aged adults, with comparatively limited representation of men and older adults. Furthermore, the trials were short-term and therefore could not assess sustained efficacy and safety, the consequences of repeated dosing, or long-term outcomes. Finally, inconsistent AE reporting across trials, including variation between TEAEs, total AEs, and limited safety summaries, precluded quantitative network synthesis of benefit with respect to harms. Moreover, limited data prevented stratification of AEs by dosage form, and reliance on pooled AE frequencies limited direct comparisons of safety profiles across interventions.

## 5 Conclusion

In this NMA, the spectrum of evidence indicates that OTC therapies demonstrate clinically meaningful efficacy for the acute management of episodic migraine compared with placebo, whereas the efficacy of some oral prescription medications varies across the outcomes. Both ibuprofen and the AAC combination demonstrated consistent benefits across two-hour pain relief and associated symptom endpoints, with the principal findings supported by both the primary fixed-effect analysis and random-effects sensitivity analyses, supporting their role as effective, accessible, and guideline-supported options for episodic migraine. Moreover, the ease of availability and relatively low cost of OTC medications further support their clinical utility. Despite the growing body of evidence on migraine therapies, there remains a paucity of data evaluating comparative safety, particularly with respect to TEAEs. Future research through larger, well-designed RCTs are needed to confirm relative benefits, define optimal dosing strategies, and evaluate long-term safety. In addition, individual-patient-data NMA are warranted to improve precision and enable more personalized management of episodic migraine.

## Data Availability

The datasets generated during the present study are available for sharing upon reasonable request to the corresponding author.

## References

[1] Headache Classification Committee of the International Headache Society (IHS). The international classification of headache disorders, 3rd edition. Cephalalgia. (2018) 38:1–211. doi: 10.1177/0333102417738202, 29368949

[2] Dong L Dong W Jin Y Jiang Y Li Z Yu D. The global burden of migraine: a 30-year trend review and future projections by age, sex, country, and region. Pain Ther. (2025) 14:297–315. doi: 10.1007/s40122-024-00690-7, 39661241 PMC11751287

[3] Cohen F Brooks CV Sun D Buse DC Reed ML Fanning KM . Prevalence and burden of migraine in the United States: a systematic review. Headache. (2024) 64:516–32. doi: 10.1111/head.14709, 38700185

[4] Lipton RB Silberstein SD. Episodic and chronic migraine headache: breaking down barriers to optimal treatment and prevention. Headache. (2015) 55:103–22. doi: 10.1111/head.12505_2, 25662743

[5] Peck J Urits I Zeien J Hoebee S Mousa M Alattar H . A comprehensive review of over-the-counter treatment for chronic migraine headaches. Curr Pain Headache Rep. (2020) 24:19. doi: 10.1007/s11916-020-00852-0, 32200435

[6] Pellesi L Do TP Hougaard A. Pharmacological management of migraine: current strategies and future directions. Expert Opin Pharmacother. (2024) 25:673–83. doi: 10.1080/14656566.2024.2349791, 38720629

[7] Ornello R Caponnetto V Ahmed F Al-Khazali HM Ambrosini A Ashina S . Evidence-based guidelines for the pharmacological treatment of migraine. Cephalalgia. (2025) 45:3331024241305381. doi: 10.1177/03331024241305381, 40277319

[8] Karlsson WK Ostinelli EG Zhuang ZA Kokoti L Christensen RH Al-Khazali HM . Comparative effects of drug interventions for the acute management of migraine episodes in adults: systematic review and network meta-analysis. BMJ. (2024) 386:e080107. doi: 10.1136/bmj-2024-080107, 39293828 PMC11409395

[9] Puledda F Younis S Huessler EM Haghdoost F Lisicki M Goadsby PJ . Efficacy, safety and indirect comparisons of lasmiditan, rimegepant, and ubrogepant for the acute treatment of migraine: a systematic review and network meta-analysis of the literature. Cephalalgia. (2023) 43:3331024231151419. doi: 10.1177/03331024231151419, 36786357

[10] Chapter 11: Undertaking network meta-analyses | Cochrane. Available online at: https://www.cochrane.org/authors/handbooks-and-manuals/handbook/current/chapter-11 (accessed Feb 9, 2026).

[11] Page MJ McKenzie JE Bossuyt PM Boutron I Hoffmann TC Mulrow CD . The PRISMA 2020 statement: an updated guideline for reporting systematic reviews. BMJ. (2021) 372:n71. doi: 10.1136/bmj.n71, 33782057 PMC8005924

[12] Sterne JAC Savović J Page MJ Elbers RG Blencowe NS Boutron I . RoB 2: a revised tool for assessing risk of bias in randomised trials. BMJ. (2019) 366:l4898. doi: 10.1136/bmj.l4898, 31462531

[13] Higgins JPT Altman DG Gøtzsche PC Jüni P Moher D Oxman AD . The Cochrane collaboration’s tool for assessing risk of bias in randomised trials. BMJ. (2011) 343:d5928. doi: 10.1136/bmj.d5928, 22008217 PMC3196245

[14] Rücker G Schwarzer G. Ranking treatments in frequentist network meta-analysis works without resampling methods. BMC Med Res Methodol. (2015) 15:58. doi: 10.1186/s12874-015-0060-8, 26227148 PMC4521472

[15] Salanti G Ades AE Ioannidis JPA. Graphical methods and numerical summaries for presenting results from multiple-treatment meta-analysis: an overview and tutorial. J Clin Epidemiol. (2011) 64:163–71. doi: 10.1016/j.jclinepi.2010.03.016, 20688472

[16] Higgins JPT Jackson D Barrett JK Lu G Ades AE White IR. Consistency and inconsistency in network meta-analysis: concepts and models for multi-arm studies. Res Synth Methods. (2012) 3:98–110. doi: 10.1002/jrsm.1044, 26062084 PMC4433772

[17] Higgins JPT Thompson SG Deeks JJ Altman DG. Measuring inconsistency in meta-analyses. BMJ. (2003) 327:557–60. doi: 10.1136/bmj.327.7414.557, 12958120 PMC192859

[18] Chaimani A Higgins JPT Mavridis D Spyridonos P Salanti G. Graphical tools for network meta-analysis in STATA. PLoS One. (2013) 8:e76654. doi: 10.1371/journal.pone.0076654, 24098547 PMC3789683

[19] Yadav R Singh TP Verma A Shukla SK. Almotriptan versus ibuprofen in migraine: a randomised placebo-controlled trial. J Indian Acad Clin Med. (2016) 17:111.

[20] Goldstein J Silberstein SD Saper JR Ryan RE Lipton RB. Acetaminophen, aspirin, and caffeine in combination versus ibuprofen for acute migraine: results from a multicenter, double-blind, randomized, parallel-group, single-dose, placebo-controlled study. Headache. (2006) 46:444–53. doi: 10.1111/j.1526-4610.2006.00376.x, 16618262

[21] Goldstein J Silberstein SD Saper JR Elkind AH Smith TR Gallagher RM . Acetaminophen, aspirin, and caffeine versus sumatriptan succinate in the early treatment of migraine: results from the ASSET trial. Headache. (2005) 45:973–82. doi: 10.1111/j.1526-4610.2005.05177.x, 16109110

[22] Lipton RB Stewart WF Ryan RE Saper J Silberstein S Sheftell F. Efficacy and safety of acetaminophen, aspirin, and caffeine in alleviating migraine headache pain: three double-blind, randomized, placebo-controlled trials. Arch Neurol. (1998) 55:210–7. doi: 10.1001/archneur.55.2.210, 9482363

[23] Lipton RB Dodick DW Ailani J Lu K Finnegan M Szegedi A . Effect of ubrogepant vs placebo on pain and the most bothersome associated symptom in the acute treatment of migraine: the ACHIEVE II randomized clinical trial. JAMA. (2019) 322:1887–98. doi: 10.1001/jama.2019.16711, 31742631 PMC6865323

[24] Voss T Lipton RB Dodick DW Dupre N Ge JY Bachman R . A phase IIb randomized, double-blind, placebo-controlled trial of ubrogepant for the acute treatment of migraine. Cephalalgia. (2016) 36:887–98. doi: 10.1177/0333102416653233, 27269043

[25] Dodick DW Lipton RB Ailani J Lu K Finnegan M Trugman JM . Ubrogepant for the treatment of migraine. N Engl J Med. (2019) 381:2230–41. doi: 10.1056/NEJMoa1813049, 31800988

[26] Yu S Kim BK Guo A Kim MH Zhang M Wang Z . Safety and efficacy of rimegepant orally disintegrating tablet for the acute treatment of migraine in China and South Korea: a phase 3, double-blind, randomised, placebo-controlled trial. Lancet Neurol. (2023) 22:476–84. doi: 10.1016/S1474-4422(23)00126-6, 37210098

[27] Croop R Goadsby PJ Stock DA Conway CM Forshaw M Stock EG . Efficacy, safety, and tolerability of rimegepant orally disintegrating tablet for the acute treatment of migraine: a randomised, phase 3, double-blind, placebo-controlled trial. Lancet. (2019) 394:737–45. doi: 10.1016/S0140-6736(19)31606-X, 31311674

[28] Lipton RB Croop R Stock EG Stock DA Morris BA Frost M . Rimegepant, an Oral calcitonin gene-related peptide receptor antagonist, for migraine. N Engl J Med. (2019) 381:142–9. doi: 10.1056/NEJMoa1811090, 31291516

[29] Marcus R Goadsby PJ Dodick D Stock D Manos G Fischer TZ. BMS-927711 for the acute treatment of migraine: a double-blind, randomized, placebo controlled, dose-ranging trial. Cephalalgia. (2014) 34:114–25. doi: 10.1177/0333102413500727, 23965396

[30] Lipton RB Thiry A Morris BA Croop R. Efficacy and safety of rimegepant 75 mg oral tablet, a CGRP receptor antagonist, for the acute treatment of migraine: a randomized, double-blind, placebo-controlled trial. J Pain Res. (2024) 17:2431–41. doi: 10.2147/JPR.S453806, 39070853 PMC11277999

[31] Misra UK Kalita J Yadav RK. Rizatriptan vs. ibuprofen in migraine: a randomised placebo-controlled trial. J Headache Pain. (2007) 8:175–9. doi: 10.1007/s10194-007-0386-7, 17563841 PMC3476139

[32] Saper J Dahlof C So Y Tfelt-Hansen P Malbecq W Loeys T . Rofecoxib in the acute treatment of migraine: a randomized controlled clinical trial. Headache. (2006) 46:264–75. doi: 10.1111/j.1526-4610.2006.00334.x, 16492236

[33] Misra UK Jose M Kalita J. Rofecoxib versus ibuprofen for acute treatment of migraine: a randomised placebo controlled trial. Postgrad Med J. (2004) 80:720–3. doi: 10.1136/pgmj.2003.012393, 15579612 PMC1743152

[34] Diener HC Bussone G de Liano H Eikermann A Englert R Floeter T . Placebo-controlled comparison of effervescent acetylsalicylic acid, sumatriptan and ibuprofen in the treatment of migraine attacks. Cephalalgia. (2004) 24:947–54. doi: 10.1111/j.1468-2982.2004.00783.x, 15482357

[35] Codispoti JR Prior MJ Fu M Harte CM Nelson EB. Efficacy of nonprescription doses of ibuprofen for treating migraine headache. A randomized controlled trial. Headache. (2001) 41:665–79. doi: 10.1046/j.1526-4610.2001.041007665.x, 11554954

[36] Sandrini G Franchini S Lanfranchi S Granella F Manzoni GC Nappi G. Effectiveness of ibuprofen-arginine in the treatment of acute migraine attacks. Int J Clin Pharmacol Res. (1998) 18:145–50. 9825271

[37] Kellstein DE Lipton RB Geetha R Koronkiewicz K Evans FT Stewart WF . Evaluation of a novel solubilized formulation of ibuprofen in the treatment of migraine headache: a randomized, double-blind, placebo-controlled, dose-ranging study. Cephalalgia. (2000) 20:233–43. doi: 10.1046/j.1468-2982.2000.00055.x, 10999673

[38] Ashina M Reuter U Smith T Krikke-Workel J Klise SR Bragg S . Randomized, controlled trial of lasmiditan over four migraine attacks: findings from the CENTURION study. Cephalalgia. (2021) 41:294–304. doi: 10.1177/0333102421989232, 33541117 PMC7961651

[39] Sakai F Takeshima T Homma G Tanji Y Katagiri H Komori M. Phase 2 randomized placebo-controlled study of lasmiditan for the acute treatment of migraine in Japanese patients. Headache. (2021) 61:755–65. doi: 10.1111/head.14122, 33990951 PMC8252620

[40] Goadsby PJ Wietecha LA Dennehy EB Kuca B Case MG Aurora SK . Phase 3 randomized, placebo-controlled, double-blind study of lasmiditan for acute treatment of migraine. Brain. (2019) 142:1894–904. doi: 10.1093/brain/awz134, 31132795 PMC6620826

[41] Kuca B Silberstein SD Wietecha L Berg PH Dozier G Lipton RB . Lasmiditan is an effective acute treatment for migraine: a phase 3 randomized study. Neurology. (2018) 91:e2222–32. doi: 10.1212/WNL.0000000000006641, 30446595 PMC6329326

[42] Färkkilä M Diener HC Géraud G Láinez M Schoenen J Harner N . Efficacy and tolerability of lasmiditan, an oral 5-HT(1F) receptor agonist, for the acute treatment of migraine: a phase 2 randomised, placebo-controlled, parallel-group, dose-ranging study. Lancet Neurol. (2012) 11:405–13. doi: 10.1016/S1474-4422(12)70047-9, 22459549

[43] Ikeda K Matsumori Y Kudo M Ishikawa T Hoshino Y Yoshimatsu H . Efficacy and safety of rimegepant for the acute treatment of migraine in Japan: a dose-ranging, double-blind, randomized controlled trial. Headache. (2025) 65:1811–20. doi: 10.1111/head.14994, 40586377 PMC12638520

[44] Johnston K Popoff E Deighton A Dabirvaziri P Harris L Thiry A . Comparative efficacy and safety of rimegepant, ubrogepant, and lasmiditan for acute treatment of migraine: a network meta-analysis. Expert Rev Pharmacoecon Outcomes Res. (2022) 22:155–66. doi: 10.1080/14737167.2021.1945444, 34148501

[45] Dodick DW. A phase-by-phase review of migraine pathophysiology. Headache. (2018) 58:4–16. doi: 10.1111/head.13300, 29697154

[46] Puledda F Silva EM Suwanlaong K Goadsby PJ. Migraine: from pathophysiology to treatment. J Neurol. (2023) 270:3654–66. doi: 10.1007/s00415-023-11706-1, 37029836 PMC10267278

[47] Ong JJY De Felice M. Migraine treatment: current acute medications and their potential mechanisms of action. Neurotherapeutics. (2018) 15:274–90. doi: 10.1007/s13311-017-0592-1, 29235068 PMC5935632

[48] Xu H Han W Wang J Li M. Network meta-analysis of migraine disorder treatment by NSAIDs and triptans. J Headache Pain. (2016) 17:113. doi: 10.1186/s10194-016-0703-0, 27957624 PMC5153398

[49] Center for Drug Evaluation and Research Summary Review. Drugs at FDA; (2019). Available online at: https://www.accessdata.fda.gov/drugsatfda_docs/nda/2020/212728Orig1s000SumR.pdf (accessed Apr 10, 2026).

[50] Deng X Zhou L Liang C Shang X Hui X Liu W . Comparison of effectiveness and safety of lasmiditan and CGRP-antagonists for the acute treatment of migraine in adults: systematic review and network meta-analysis of randomised trials. J Headache Pain. (2024) 25:16. doi: 10.1186/s10194-024-01723-4, 38311738 PMC10840250

[51] Lipton RB Hamelsky SW Dayno JM. What do patients with migraine want from acute migraine treatment? Headache. (2002) 42:3–9. doi: 10.1046/j.1526-4610.2002.0420s1003.x, 11966858

[52] Ailani J Burch RC Robbins MS Board of Directors of the American Headache Society. The American headache society consensus statement: update on integrating new migraine treatments into clinical practice. Headache. (2021) 61:1021–39. doi: 10.1111/head.14153, 34160823

[53] Nguyen JL Munshi K Peasah SK Swart ECS Kohli M Henderson R . Trends in utilization and costs of migraine medications, 2017–2020. J Headache Pain. (2022) 23:111. doi: 10.1186/s10194-022-01476-y, 36031609 PMC9420279

[54] Abdi YH Abdi MS Bashir SG Ahmed NI Abdullahi YB. Understanding Global Health inequality and inequity: causes, consequences, and the path toward justice in healthcare. Public Health Chall. (2025) 4:e70156. doi: 10.1002/puh2.70156, 41146749 PMC12553980

[55] Ghinea N. The increasing costs of medicines and their implications for patients, physicians and the health system. Intern Med J. (2024) 54:545–50. doi: 10.1111/imj.16370, 38572698

[56] Vincent Rajkumar S. The high cost of prescription drugs: causes and solutions. Blood Cancer J. (2020) 10:71. doi: 10.1038/s41408-020-0338-x, 32576816 PMC7311400

[57] Woods B Palacios A Sculpher M. A framework for using cost-effectiveness analysis to support pricing and reimbursement decisions for new Pharmaceuticals in a Context of evolving treatments, prices, and evidence. PharmacoEconomics. (2025) 43:363–73. doi: 10.1007/s40273-024-01450-3, 39739218 PMC12227514

