# Supplementary material for: Comparative efficacy of AAC and ibuprofen versus lasmiditan, ubrogepant, and rimegepant for the acute treatment of episodic migraine in adults: a systematic review and network meta-analysis
Source: Front Neurol. 2026 Sep 15;17:1884713. doi: 10.3389/fneur.2026.1884713 (PMC13619513; doi:10.3389/fneur.2026.1884713)
Supplement: Supplementary file 1 [file Supplementary_file_1.docx]

# **Comparative Efficacy of AAC and Ibuprofen Versus Lasmiditan, Ubrogepant, and Rimegepant for the Acute Treatment of Episodic Migraine in Adults: A Systematic Review and Network Meta-analysis**

# **Supplementary Materials**

Table S1: PRISMA NMA Checklist of Items to Include When Reporting a Systematic Review Involving a NMA

Table S2: Search strategy

Figure S1: Risk of bias assessment of included studies

Figure S2: Summary Plot Risk of bias assessment

Table S3. League table of pain relief at 2h

Figure S3. Comparison-adjusted funnel plots of pain relief at two-hour post dose

Table S4. League table of pain freedom at 2h

Figure S4. Comparison-adjusted funnel plots of pain freedom at two-hour post dose

Table S5. League table of photophobia at 2h

Table S6. League table of phonophobia at 2h

Table S7. League table of Nausea at 2h

Figure S5. Sensitivity analysis of the outcomes using random effect model

Table S8: Treatment-emergent adverse events (TEAEs)

**Figures and tables**

Table S1: PRISMA NMA Checklist of Items to Include When Reporting a Systematic Review Involving a Network Meta-analysis

| **Section/Topic** | **Item #** | **Checklist Item** | **Reported on Page #** |
| --- | --- | --- | --- |
| **TITLE** |  |  |  |
| Title | 1 | Identify the report as a systematic review *incorporating a network meta-analysis (or related form of meta-analysis).* | Along with abstract |
|  |  |  |  |
| **ABSTRACT** |  |  |  |
| Structured summary | 2 | Provide a structured summary including, as applicable:  **Background:** main objectives  **Methods:** data sources; study eligibility criteria, participants, and interventions; study appraisal; and *synthesis methods, such as network meta-analysis.*  **Results:** number of studies and participants identified; summary estimates with corresponding confidence/credible intervals; *treatment rankings may also be discussed. Authors may choose to summarize pairwise comparisons against a chosen treatment included in their analyses for brevity.*  **Discussion/Conclusions:** limitations; conclusions and implications of findings.  **Other:** primary source of funding; systematic review registration number with registry name. | Abstract |
|  |  |  |  |
| **INTRODUCTION** |  |  |  |
| Rationale | 3 | Describe the rationale for the review in the context of what is already known*, including mention of why a network meta-analysis has been conducted.* | Introduction |
| Objectives | 4 | Provide an explicit statement of questions being addressed, with reference to participants, interventions, comparisons, outcomes, and study design (PICOS). | Introduction |
|  |  |  |  |
| **METHODS** |  |  |  |
| Protocol and registration | 5 | Indicate whether a review protocol exists and if and where it can be accessed (e.g., Web address); and, if available, provide registration information, including registration number. | Methods |
| Eligibility criteria | 6 | Specify study characteristics (e.g., PICOS, length of follow-up) and report characteristics (e.g., years considered, language, publication status) used as criteria for eligibility, giving rationale. *Clearly describe eligible treatments included in the treatment network, and note whether any have been clustered or merged into the same node (with justification).* | Methods |
| Information sources | 7 | Describe all information sources (e.g., databases with dates of coverage, contact with study authors to identify additional studies) in the search and date last searched. | Methods |
| Search | 8 | Present full electronic search strategy for at least one database, including any limits used, such that it could be repeated. | Table S2 |
| Study selection | 9 | State the process for selecting studies (i.e., screening, eligibility, included in systematic review, and, if applicable, included in the meta-analysis). | Methods |
| Data collection process | 10 | Describe method of data extraction from reports (e.g., piloted forms, independently, in duplicate) and any processes for obtaining and confirming data from investigators. | Methods |
| Data items | 11 | List and define all variables for which data were sought (e.g., PICOS, funding sources) and any assumptions and simplifications made. | Methods |
| **Geometry of the network** | **S1** | Describe methods used to explore the geometry of the treatment network under study and potential biases related to it. This should include how the evidence base has been graphically summarized for presentation, and what characteristics were compiled and used to describe the evidence base to readers. | Methods |
| Risk of bias within individual studies | 12 | Describe methods used for assessing risk of bias of individual studies (including specification of whether this was done at the study or outcome level), and how this information is to be used in any data synthesis. | Methods |
| Summary measures | 13 | State the principal summary measures (e.g., risk ratio, difference in means). *Also describe the use of additional summary measures assessed, such as treatment rankings and surface under the cumulative ranking curve (SUCRA) values, as well as modified approaches used to present summary findings from meta-analyses.* | Methods |
| Planned methods of analysis | 14 | Describe the methods of handling data and combining results of studies for each network meta-analysis. This should include, but not be limited to:   - *Handling of multi-arm trials;* - *Selection of variance structure;* - *Selection of prior distributions in Bayesian analyses; and* - *Assessment of model fit.* | Methods |
| **Assessment of Inconsistency** | **S2** | Describe the statistical methods used to evaluate the agreement of direct and indirect evidence in the treatment network(s) studied. Describe efforts taken to address its presence when found. | Methods |
| Risk of bias across studies | 15 | Specify any assessment of risk of bias that may affect the cumulative evidence (e.g., publication bias, selective reporting within studies). | Methods |
| Additional analyses | 16 | Describe methods of additional analyses if done, indicating which were pre-specified. This may include, but not be limited to, the following:   - Sensitivity or subgroup analyses; - Meta-regression analyses; - *Alternative formulations of the treatment network; and* - *Use of alternative prior distributions for Bayesian analyses (if applicable).* | NA |
|  |  |  |  |
| **RESULTS†** |  |  |  |
| Study selection | 17 | Give numbers of studies screened, assessed for eligibility, and included in the review, with reasons for exclusions at each stage, ideally with a flow diagram. | Result |
| **Presentation of network structure** | **S3** | Provide a network graph of the included studies to enable visualization of the geometry of the treatment network. | Result, Figure 2 a, b, c, d and e |
| **Summary of network geometry** | **S4** | Provide a brief overview of characteristics of the treatment network. This may include commentary on the abundance of trials and randomized patients for the different interventions and pairwise comparisons in the network, gaps of evidence in the treatment network, and potential biases reflected by the network structure. | Result |
| Study characteristics | 18 | For each study, present characteristics for which data were extracted (e.g., study size, PICOS, follow-up period) and provide the citations. | Result, Table 1 |
| Risk of bias within studies | 19 | Present data on risk of bias of each study and, if available, any outcome level assessment. | Result, S1a, b and S2 |
| Results of individual studies | 20 | For all outcomes considered (benefits or harms), present, for each study: 1) simple summary data for each intervention group, and 2) effect estimates and confidence intervals. *Modified approaches may be needed to deal with information from larger networks.* | Result |
| Synthesis of results | 21 | Present results of each meta-analysis done, including confidence/credible intervals. *In larger networks, authors may focus on comparisons versus a particular comparator (e.g. placebo or standard care), with full findings presented in an appendix. League tables and forest plots may be considered to summarize pairwise comparisons.* If additional summary measures were explored (such as treatment rankings), these should also be presented. | Result |
| **Exploration for inconsistency** | **S5** | Describe results from investigations of inconsistency. This may include such information as measures of model fit to compare consistency and inconsistency models, *P* values from statistical tests, or summary of inconsistency estimates from different parts of the treatment network. | Result |
| Risk of bias across studies | 22 | Present results of any assessment of risk of bias across studies for the evidence base being studied. | Result |
| Results of additional analyses | 23 | Give results of additional analyses, if done (e.g., sensitivity or subgroup analyses, meta-regression analyses*, alternative network geometries studied, alternative choice of prior distributions for Bayesian analyses,* and so forth). | NA |
|  |  |  |  |
| **DISCUSSION** |  |  |  |
| Summary of evidence | 24 | Summarize the main findings, including the strength of evidence for each main outcome; consider their relevance to key groups (e.g., healthcare providers, users, and policy-makers). | Discussion |
| Limitations | 25 | Discuss limitations at study and outcome level (e.g., risk of bias), and at review level (e.g., incomplete retrieval of identified research, reporting bias). *Comment on the validity of the assumptions, such as transitivity and consistency. Comment on any concerns regarding network geometry (e.g., avoidance of certain comparisons).* | Discussion |
| Conclusions | 26 | Provide a general interpretation of the results in the context of other evidence, and implications for future research. | Discussion |
|  |  |  |  |
| **FUNDING** |  |  |  |
| Funding | 27 | Describe sources of funding for the systematic review and other support (e.g., supply of data); role of funders for the systematic review. This should also include information regarding whether funding has been received from manufacturers of treatments in the network and/or whether some of the authors are content experts with professional conflicts of interest that could affect use of treatments in the network. | Under the section funding |

PICOS = population, intervention, comparators, outcomes, study design.

* Text in italics indicateS wording specific to reporting of network meta-analyses that has been added to guidance from the PRISMA statement.

† Authors may wish to plan for use of appendices to present all relevant information in full detail for items in this section.

**Table S2: Search strategy**

| **Search query** | **Search string_ PubMed** | **Hits** |
| --- | --- | --- |
| #1 | ((((acetylsalicylic acid[MeSH Terms]) OR ("acetylsalicylic acid"[Title/Abstract] OR acylpyrin[Title/Abstract] OR aloxiprimum[Title/Abstract] OR colfarit[Title/Abstract] OR dispril[Title/Abstract] OR easprin[Title/Abstract] OR ecotrin[Title/Abstract] OR endosprin[Title/Abstract] OR magnecyl[Title/Abstract] OR micristin[Title/Abstract] OR polopirin[Title/Abstract] OR polopiryna[Title/Abstract] OR solprin[Title/Abstract] OR solupsan[Title/Abstract] OR zorprin[Title/Abstract] OR acetysal[Title/Abstract] OR "ASA"[Title/Abstract] OR "acetyl-salicylic acid"[Title/Abstract] OR aspirin[Title/Abstract])) AND ((paracetamol OR acetaminophen[MeSH Terms]) OR (acetaminophen[Title/Abstract] OR paracetamol[Title/Abstract] OR tylenol[Title/Abstract] OR panadol[Title/Abstract] OR apap[Title/Abstract] OR crocin[Title/Abstract] OR dolex[Title/Abstract]))) AND ((caffeine[MeSH Terms]) OR (caffeine[Title/Abstract] OR vivarin[Title/Abstract] OR caffedrine[Title/Abstract] OR "coffeinum n"[Title/Abstract] OR percutaféine[Title/Abstract] OR dexitac[Title/Abstract] OR durvitan[Title/Abstract] OR "no doz"[Title/Abstract] OR "percoffedrinol n"[Title/Abstract] OR "quick-pep"[Title/Abstract] OR "quick pep"[Title/Abstract] OR quickpep[Title/Abstract] OR "coffeinum purrum"[Title/Abstract]))) | 352 |
| #2 | (ibuprofen[MeSH Terms]) OR (ibuprofen[Title/Abstract] OR motrin[Title/Abstract] OR brufen[Title/Abstract]) | 19251 |
| #3 | (Lasmiditan[Supplementary Concept] OR (lasmiditan[Title/Abstract] OR reyvow[Title/Abstract] OR "col-144"[Title/Abstract] OR "ly573144"[Title/Abstract]) | 223 |
| #4 | (Ubrogepant[Supplementary Concept] OR (ubrogepant[Title/Abstract] OR ubrogépant[Title/Abstract] OR ubrogepantum[Title/Abstract] OR ubrelvy[Title/Abstract] OR "mk-1602"[Title/Abstract]) | 184 |
| #5 | (rimegepant[Supplementary Concept]) OR ("rimegepant sulfate"[Supplementary Concept]) OR (rimegepant[Title/Abstract] OR "nurtec odt"[Title/Abstract] OR "bms-927711"[Title/Abstract] OR "bhv 3000"[Title/Abstract] OR "bhv3000"[Title/Abstract] OR "bms 927711"[Title/Abstract] OR "bms927711"[Title/Abstract] OR "vydura"[Title/Abstract]) | 258 |
| #6 | (Migraine[MeSH Terms]) OR (headache[MeSH Terms]) OR ("tension-type headache"[Title/Abstract] OR "tension type headache"[Title/Abstract] OR "tension headache"[Title/Abstract] OR "TTH"[Title/Abstract] OR headache*[Title/Abstract] OR "head pain"[Title/Abstract] OR migraine[Title/Abstract] OR cephalalgia*[Title/Abstract] OR cephalgia[Title/Abstract] OR cephalodynia[Title/Abstract] OR "cranial pain"[Title/Abstract] OR hemicrania[Title/Abstract] OR migrainosus[Title/Abstract] OR migrainous[Title/Abstract] OR hemicranicus[Title/Abstract] OR "tension-headache"[Title/Abstract]) | 151265 |
| #7 | ("clinical trial" OR "'single blind procedure" OR "double blind procedure" OR "clinical study" OR "randomized controlled trial" OR "controlled study" OR "crossover procedure" OR "crossover-trial" OR cohort OR "systematic review"[Publication Type]) OR ("clinical trial" "clinical-trial*"[Title/Abstract] OR random*[Title/Abstract] OR factorial*[Title/Abstract] OR crossover*[Title/Abstract] OR assign*[Title/Abstract] OR allocat*[Title/Abstract] OR "clinical trial"[Title/Abstract] OR "clinical trials"[Title/Abstract] OR "intervention study"[Title/Abstract] OR "intervention studies"[Title/Abstract] OR "case control"[Title/Abstract] OR cohort[Title/Abstract] OR longitudinal*[Title/Abstract] OR prospective[Title/Abstract] OR prospectively[Title/Abstract] OR retrospective[Title/Abstract] OR "comparative study"[Title/Abstract] OR "comparative studies"[Title/Abstract] OR "systematic review"[Title/Abstract] OR "meta-analysis"[Title/Abstract] OR "meta-analyses"[Title/Abstract] OR review*[Title/Abstract] OR childhood[Title/Abstract] OR children[Title/Abstract] OR child[Title/Abstract] OR adolescen*[Title/Abstract] OR teen[Title/Abstract] OR teens[Title/Abstract] OR teenager*[Title/Abstract] OR girl[Title/Abstract] OR girls[Title/Abstract] OR boy[Title/Abstract] OR boys[Title/Abstract] OR paediatric*[Title/Abstract] OR pediatric*[Title/Abstract] OR juvenil*[Title/Abstract] OR infant[Title/Abstract] OR infants[Title/Abstract] OR "young people"[Title/Abstract] OR "young person"[Title/Abstract] OR "young adult"[Title/Abstract] OR "young m*n"[Title/Abstract] OR "young wom*n"[Title/Abstract] OR "schoolchild*"[Title/Abstract] OR clinical[Title/Abstract] OR volunteer*[Title/Abstract] OR subject*[Title/Abstract] OR human*[Title/Abstract] OR patient*[Title/Abstract] OR trial*[Title/Abstract] OR adult*[Title/Abstract] OR kid[Title/Abstract] OR kids[Title/Abstract] OR pregnant[Title/Abstract] OR pregnancy[Title/Abstract] OR babies[Title/Abstract] OR baby[Title/Abstract] OR babyhood[Title/Abstract] OR neonate[Title/Abstract] OR neonates[Title/Abstract] OR neonatal[Title/Abstract] OR neonatus[Title/Abstract] OR newborn[Title/Abstract] OR newborns[Title/Abstract] OR "new-born"[Title/Abstract] OR "new-borns"[Title/Abstract] OR perinatal[Title/Abstract] OR preschool[Title/Abstract] OR preschools[Title/Abstract] OR preschooler[Title/Abstract] OR preschoolers[Title/Abstract] OR "pre-school"[Title/Abstract] OR "pre-schools"[Title/Abstract] OR "pre-schooler"[Title/Abstract] OR "pre-schoolers"[Title/Abstract] OR puberty[Title/Abstract] OR pubertal[Title/Abstract] OR puberta[Title/Abstract] OR pubertas[Title/Abstract] OR pubescent[Title/Abstract] OR pubescence[Title/Abstract] OR "school child"[Title/Abstract] OR "school children"[Title/Abstract] OR schoolchild[Title/Abstract] OR schoolchildren[Title/Abstract] OR toddler[Title/Abstract] OR toddlers[Title/Abstract] OR youth[Title/Abstract] OR youths[Title/Abstract] OR aged[Title/Abstract] OR geriatric[Title/Abstract] OR geriatrics[Title/Abstract] OR elder[Title/Abstract] OR elderly[Title/Abstract] OR elders[Title/Abstract] OR "older population"[Title/Abstract] OR "older populations"[Title/Abstract] OR frail[Title/Abstract] OR man[Title/Abstract] OR men[Title/Abstract] OR women[Title/Abstract] OR woman[Title/Abstract] OR "homo sapiens"[Title/Abstract] OR hominidae[Title/Abstract] OR male[Title/Abstract] OR males[Title/Abstract] OR female[Title/Abstract] OR females[Title/Abstract] OR healthy[Title/Abstract]) | 20569938 |
| #8 | preclinical[Title] OR "pre clinical"[Title] OR "pre-clinical"[Title] OR "nonclinical"[Title] OR "non clinical"[Title] OR "non-clinical"[Title] OR "in vitro"[Title] OR "in-vitro"[Title] OR "in vivo"[Title] OR "in-vivo"[Title] OR animal*[Title] OR avian[Title] OR baboon[Title] OR bird[Title] OR canine[Title] OR cat[Title] OR chicken[Title] OR dog[Title] OR dogs[Title] OR feline[Title] OR fish[Title] OR macaca[Title] OR macaque[Title] OR mice[Title] OR monkey[Title] OR mouse[Title] OR murine[Title] OR octopus[Title] OR bovine[Title] OR guinea[Title] OR pig[Title] OR pigs[Title] OR pigeon[Title] OR porcine[Title] OR rabbit[Title] OR rat[Title] OR rats[Title] OR rhesus[Title] OR rodent[Title] OR hamster[Title] OR sheep[Title] OR "nonhuman"[Title] OR "animal experiment"[Title] OR "animal model"[Title] OR "animal tissue"[Title] OR "human cell"[Title] OR "human tissue"[Title] OR "rabbit"[Title] OR "animal cell"[Title] OR "animal"[Title] OR "wistar rat*"[Title] OR horses[Title] | 3035130 |
| #9 | ((#1 AND #6 AND #7) NOT #8) AND (1990/1/1:2025/7/31[pdat]) | 81 |
| #10 | ((#2 AND #6 AND #7) NOT #8) AND (1990/1/1:2025/7/31[pdat]) | 451 |
| #11 | ((#3 AND #6 AND #7) NOT #8) AND (1990/1/1:2025/7/31[pdat]) | 179 |
| #12 | ((#4 AND #6 AND #7) NOT #8) AND (1990/1/1:2025/7/31[pdat]) | 159 |
| #13 | ((#5 AND #6 AND #7) NOT #8) AND (1990/1/1:2025/7/31[pdat]) | 197 |
| #14 | (#9 OR #10 OR #11 OR #12 OR #13) AND (1990/1/1:2025/7/31[pdat]) | 890 |
| **Search query** | **Search string_ Embase** | **Hits** |
| #1 | ('acetylsalicylic acid'/exp/mj OR 'acetylsalicylic acid':ab,ti OR acylpyrin:ab,ti OR aloxiprimum:ab,ti OR colfarit:ab,ti OR dispril:ab,ti OR easprin:ab,ti OR ecotrin:ab,ti OR endosprin:ab,ti OR magnecyl:ab,ti OR micristin:ab,ti OR polopirin:ab,ti OR polopiryna:ab,ti OR solprin:ab,ti OR solupsan:ab,ti OR zorprin:ab,ti OR acetysal:ab,ti OR 'asa':ab,ti OR 'acetyl-salicylic acid':ab,ti OR aspirin:ab,ti) AND ('paracetamol'/exp/mj OR acetaminophen:ab,ti OR paracetamol:ab,ti OR tylenol:ab,ti OR panadol:ab,ti OR apap:ab,ti OR crocin:ab,ti OR dolex:ab,ti) AND ('caffeine'/exp/mj OR caffeine:ab,ti OR vivarin:ab,ti OR caffedrine:ab,ti OR 'coffeinum n':ab,ti OR percutaféine:ab,ti OR dexitac:ab,ti OR durvitan:ab,ti OR 'no doz':ab,ti OR 'percoffedrinol n':ab,ti OR 'quick-pep':ab,ti OR 'quick pep':ab,ti OR quickpep:ab,ti OR 'coffeinum purrum':ab,ti) | 793 |
| #2 | ibuprofen'/exp/mj OR ibuprofen:ab,ti OR mortin:ab,ti OR brufen:ab,ti OR 'ibu':ab,ti | 31594 |
| #3 | lasmiditan'/exp/mj OR lasmiditan:ab,ti OR r?yvow:ab,ti OR 'col-144':ab,ti OR ly573144:ab,ti | 417 |
| #4 | ubrogepant'/exp/mj OR ubrogepant:ab,ti OR ubrogépant:ab,ti OR ubrogepantum:ab,ti OR ubrelvy:ab,ti OR 'mk-1602':ab,ti | 388 |
| #5 | rimegepant'/exp/mj OR rimegepant:ab,ti OR 'nurtec odt':ab,ti OR 'bms-927711':ab,ti OR 'bhv 3000':ab,ti OR 'bhv3000':ab,ti OR 'bms 927711':ab,ti OR 'bms927711':ab,ti OR 'vydura':ab,ti | 591 |
| #6 | migraine'/exp OR 'headache'/exp OR 'tension headache'/exp OR 'tension-type headache':ab,ti OR 'tension type headache':ab,ti OR 'tension headache':ab,ti OR 'tth':ab,ti OR headache*:ab,ti OR 'head pain':ab,ti OR migraine:ab,ti OR cephalalgia*:ab,ti OR cephalgia:ab,ti OR cephalodynia:ab,ti OR 'cranial pain':ab,ti OR hemicrania:ab,ti OR migrainosus:ab,ti OR migrainous:ab,ti OR hemicranicus:ab,ti OR 'tension-headache':ab,ti | 425806 |
| #7 | clinical trial'/exp OR 'clinical trial' OR 'clinical trial (topic)'/exp OR 'clinical trial (topic)' OR 'single blind procedure'/exp OR 'single blind procedure' OR 'double blind procedure'/exp OR 'double blind procedure' OR 'triple blind procedure'/exp OR 'triple blind procedure' OR 'clinical study'/exp OR 'clinical study' OR 'randomized controlled trial'/exp OR 'randomized controlled trial' OR 'controlled study'/exp OR 'controlled study' OR 'randomized controlled trial (topic)'/exp OR 'randomized controlled trial (topic)' OR 'controlled clinical trial'/exp OR 'controlled clinical trial' OR 'crossover procedure'/exp OR 'crossover procedure' OR 'crossover procedure topic'/exp OR 'crossover procedure topic' OR 'evaluation study'/exp OR 'evaluation study' OR 'cohort analysis'/exp OR 'cohort analysis' OR 'cohort analysis topic'/exp OR 'cohort analysis topic' OR 'comparative effectiveness'/exp OR 'comparative effectiveness' OR 'comparative study'/exp OR 'comparative study' OR 'evidence based medicine'/exp OR 'evidence based medicine' OR 'case study'/exp OR 'case study' OR 'systematic review'/exp OR 'systematic review' OR 'meta analysis'/exp OR 'meta analysis' OR random*:ab,ti OR factorial*:ab,ti OR crossover*:ab,ti OR ((cross NEAR/1 over*):ab,ti) OR placebo*:ab,ti OR ((doubl* NEAR/1 blind*):ab,ti) OR ((singl* NEAR/1 blind*):ab,ti) OR assign*:ab,ti OR allocat*:ab,ti OR 'clinical trial':ti,ab OR 'clinical trials':ti,ab OR 'intervention study':ab,ti OR 'intervention studies':ab,ti OR 'case control':ab,ti OR cohort:ab,ti OR longitudinal*:ab,ti OR prospective:ab,ti OR prospectively:ab,ti OR retrospective:ab,ti OR 'comparative study':ab,ti OR 'comparative studies':ab,ti OR 'systematic review':ab,ti OR 'meta-analysis':ab,ti OR 'meta-analyses':ab,ti OR review*:ab,ti OR childhood:ab,ti OR children:ab,ti OR child:ab,ti OR adolescen*:ab,ti OR teen:ab,ti OR teens:ab,ti OR teenager*:ab,ti OR girl:ab,ti OR girls:ab,ti OR boy:ab,ti OR boys:ab,ti OR p?ediatric?:ab,ti OR juvenil*:ab,ti OR infant:ab,ti OR infants:ab,ti OR 'young people':ab,ti OR 'young person':ab,ti OR 'young adult':ab,ti OR 'young m?n':ab,ti OR 'young wom?n':ab,ti OR 'schoolchild*':ab,ti OR clinical:ab,ti OR volunteer*:ab,ti OR subject*:ab,ti OR human*:ab,ti OR patient*:ab,ti OR trial*:ab,ti OR adult*:ab,ti OR kid*:ab,ti OR pregnant:ab,ti OR pregnancy:ab,ti OR babies:ab,ti OR baby:ab,ti OR babyhood:ab,ti OR neonate:ab,ti OR neonates:ab,ti OR neonatal:ab,ti OR neonatus:ab,ti OR newborn:ab,ti OR newborns:ab,ti OR 'new-born':ab,ti OR 'new-borns':ab,ti OR perinatal:ab,ti OR preschool:ab,ti OR preschools:ab,ti OR preschooler:ab,ti OR preschoolers:ab,ti OR 'pre-school':ab,ti OR 'pre-schools':ab,ti OR 'pre-schooler':ab,ti OR 'pre-schoolers':ab,ti OR puberty:ab,ti OR pubertal:ab,ti OR puberta:ab,ti OR pubertas:ab,ti OR pubescent:ab,ti OR pubescence:ab,ti OR 'school child':ab,ti OR 'school children':ab,ti OR schoolchild:ab,ti OR schoolchildren:ab,ti OR toddler:ab,ti OR toddlers:ab,ti OR youth:ab,ti OR youths:ab,ti OR aged:ab,ti OR geriatric:ab,ti OR geriatrics:ab,ti OR elder:ab,ti OR elderly:ab,ti OR elders:ab,ti OR 'older population':ab,ti OR 'older populations':ab,ti OR frail:ab,ti OR man:ab,ti OR men:ab,ti OR women:ab,ti OR woman:ab,ti OR 'homo sapiens':ab,ti OR hominidae:ab,ti OR male:ab,ti OR males:ab,ti OR female:ab,ti OR females:ab,ti OR healthy:ab,ti | 32929945 |
| #8 | preclinical:ti OR 'pre clinical':ti OR 'pre-clinical':ti OR 'nonclinical':ti OR 'non clinical':ti OR 'non-clinical':ti OR 'in vitro':ti OR 'in-vitro':ti OR 'in vivo':ti OR 'in-vivo':ti OR animal*:ti OR avian:ti OR baboon:ti OR bird:ti OR canine:ti OR cat:ti OR chicken:ti OR dog:ti OR dogs:ti OR feline:ti OR fish:ti OR macaca:ti OR macaque:ti OR mice:ti OR monkey:ti OR mouse:ti OR murine:ti OR octopus:ti OR bovine:ti OR guinea:ti OR pig:ti OR pigs:ti OR pigeon:ti OR porcine:ti OR rabbit:ti OR rat:ti OR rats:ti OR rhesus:ti OR rodent:ti OR hamster:ti OR sheep:ti OR 'nonhuman':ti OR 'animal experiment':ti OR 'animal model':ti OR 'animal tissue':ti OR 'human cell':ti OR 'human tissue':ti OR 'rabbit':ti OR 'animal cell':ti OR 'animal':ti OR 'wistar rat*':ti OR horses:ti | 3607521 |
| #9 | ((#1 AND #6 AND #7) NOT #8) AND ([embase]/lim OR [medline]/lim OR [preprint]/lim OR [pubmed-not-medline]/lim) AND [1990-2025]/py | 156 |
| #10 | ((#2 AND #6 AND #7) NOT #8) AND ([embase]/lim OR [medline]/lim OR [preprint]/lim OR [pubmed-not-medline]/lim) AND [1990-2025]/py | 1378 |
| #11 | ((#3 AND #6 AND #7) NOT #8) AND ([embase]/lim OR [medline]/lim OR [preprint]/lim OR [pubmed-not-medline]/lim) AND [1990-2025]/py | 320 |
| #12 | ((#4 AND #6 AND #7) NOT #8) AND ([embase]/lim OR [medline]/lim OR [preprint]/lim OR [pubmed-not-medline]/lim) AND [1990-2025]/py | 342 |
| #13 | ((#5 AND #6 AND #7) NOT #8) AND ([embase]/lim OR [medline]/lim OR [preprint]/lim OR [pubmed-not-medline]/lim) AND [1990-2025]/py | 486 |
| #14 | (#9 OR #10 OR #11 OR #12 OR #13) AND ([embase]/lim OR [medline]/lim OR [preprint]/lim OR [pubmed-not-medline]/lim) AND [1990-2025]/py | 2393 |
| **Search query** | **Search string_ Cochrane** | **Hits** |
| #1 | MeSH descriptor: [Aspirin] explode all trees | 7987 |
| #2 | (“acetylsalicylic acid” OR acylpyrin OR aloxiprimum OR colfarit OR dispril OR easprin OR ecotrin OR endosprin OR magnecyl OR micristin OR polopirin OR polopiryna OR solprin OR solupsan OR zorprin OR acetysal OR “ASA” OR “acetyl-salicylic acid” OR aspirin):ti,ab,kw | 60290 |
| #3 | MeSH descriptor: [Acetaminophen] explode all trees | 4077 |
| #4 | (acetaminophen OR paracetamol OR tylenol OR panadol OR "apap" OR crocin OR dolex):ti,ab,kw | 14317 |
| #5 | MeSH descriptor: [Caffeine] explode all trees | 2931 |
| #6 | (caffeine OR vivarin OR caffedrine OR “coffeinum n” OR percutaféine OR dexitac OR durvitan OR “no doz” OR “percoffedrinol n” OR “quick-pep” OR “quick pep” OR quickpep OR “coffeinum purrum”):ti,ab,kw | 6161 |
| #7 | ((#1 OR #2) AND (#3 OR #4) AND (#5 OR #6)) | 95 |
| #8 | MeSH descriptor: [Ibuprofen] explode all trees | 2556 |
| #9 | (ibuprofen OR motrin OR brufen):ti,ab,kw | 6146 |
| #10 | #8 OR #9 | 6146 |
| #11 | (lasmiditan OR reyvow OR "col-144" OR "ly573144"):ti,ab,kw | 161 |
| #12 | (ubrogepant OR ubrogépant OR ubrogepantum OR ubrelvy OR "mk-1602"):ti,ab,kw | 144 |
| #13 | (Rimegepant OR "nurtec odt" OR "bms-927711" OR "bhv 3000" OR "bhv3000" OR "bms 927711" OR "bms927711" OR "vydura"):ti,ab,kw | 179 |
| #14 | MeSH descriptor: [Migraine Disorders] explode all trees | 3944 |
| #15 | ("tension-type headache" OR "tension type headache" OR "tension headache" OR "TTH" OR headache* OR "head pain" OR migraine OR cephalalgia* OR cephalgia OR cephalodynia OR "cranial pain" OR hemicrania OR migrainosus OR migrainous OR hemicranicus OR "tension-headache"):ti,ab,kw | 43796 |
| #16 | #14 OR #15 | 43796 |
| #17 | [mh “clinical study”] OR [mh “clinical trial”] OR [mh “randomized controlled trial”] OR [mh “single-blind method”] OR [mh “Double-blind method”] OR [mh “controlled clinical trial”] OR [mh “clinical trial as topic”] OR [mh “randomized controlled trial as topic”] OR [mh “crossover design”] OR [mh “cohort study”] OR [mh “comparative study”] OR [mh “Evidence Based Medicine”] OR (((cross NEAR/2 over*) OR (doubl* NEAR/1 blind*) OR (singl* NEAR/1 blind*)) OR random* OR factorial* OR crossover* OR placebo* OR assign* OR allocat* OR “clinical trial” OR “clinical trials” OR “intervention study” OR “intervention studies” OR “case control” OR cohort OR longitudinal* OR prospective OR prospectively OR retrospective OR “comparative study” OR “comparative studies” OR “systematic review” OR “meta-analysis” OR “meta-analyses” OR review* OR childhood OR children OR child OR adolescen* OR teen OR teens OR teenager* OR girl OR girls OR boy OR boys OR p?ediatric? OR juvenil* OR infant OR infants OR “young people” OR “young person” OR “young adult” OR “young m?n” OR “young wom?n” OR “schoolchild*” OR clinical OR volunteer* OR subject* OR human* OR patient* OR trial* OR adult* OR kid* OR pregnant OR pregnancy OR babies OR baby OR babyhood OR neonate OR neonates OR neonatal OR neonatus OR newborn OR newborns OR “new-born” OR “new-borns” OR perinatal OR preschool OR preschools OR preschooler OR preschoolers OR “pre-school” OR “pre-schools” OR “pre-schooler” OR “pre-schoolers” OR puberty OR pubertal OR puberta OR pubertas OR pubescent OR pubescence OR “school child” OR “school children” OR schoolchild OR schoolchildren OR toddler OR toddlers OR youth OR youths OR aged OR geriatric OR geriatrics OR elder OR elderly OR elders OR “older population” OR “older populations” OR frail OR man OR men OR women OR woman OR “homo sapiens” OR hominidae OR male OR males OR female OR females OR healthy OR "randomi?ed controlled trial"):ti,ab,kw | 2161306 |
| #18 | (#7 AND #16 AND #17) with Publication Year from 1990 to 2025, in Trials | 56 |
| #19 | (#10 AND #16 AND #17) with Publication Year from 1990 to 2025, in Trials | 379 |
| #20 | (#11 AND #16 AND #17) with Publication Year from 1990 to 2025, in Trials | 145 |
| #21 | (#12 AND #16 AND #17) with Publication Year from 1990 to 2025, in Trials | 139 |
| #22 | (#13 AND #16 AND #17) with Publication Year from 1990 to 2025, in Trials | 174 |

**Figure S1: Risk of bias assessment of included studies**

**Figure S1a: Rob 2 for parallel trials**

**Figure S1b: Rob 2 for crossover trials**

**Figure S2: Summary Plot Risk of bias assessment**

**Table S3. League table of pain relief at 2h**

League table presenting pairwise relative effects across treatments (Risk Ratio with 95% CI), where each cell compares the treatment in the column against the treatment in the row. The upper‑right triangle shows column‑versus‑row estimates, and the lower‑left triangle shows the corresponding row‑versus‑column reciprocal comparisons. “#” indicate the FDA approved doses.

Figure S3. Comparison-adjusted funnel plots of pain relief at two-hour post dose. “#” indicate the FDA approved doses.

**Table S4. League table of pain freedom at 2h**

League table presenting pairwise relative effects across treatments (Risk Ratio with 95% CI), where each cell compares the treatment in the column against the treatment in the row. The upper‑right triangle shows column‑versus‑row estimates, and the lower‑left triangle shows the corresponding row‑versus‑column reciprocal comparisons. “#” indicate the FDA approved doses.

Figure S4. Comparison-adjusted funnel plots of pain freedom at two-hour post dose. “#” indicate the FDA approved doses.

**Table S5. League table of photophobia at 2h**

League table presenting pairwise relative effects across treatments (Risk Ratio with 95% CI), where each cell compares the treatment in the column against the treatment in the row. The upper‑right triangle shows column‑versus‑row estimates, and the lower‑left triangle shows the corresponding row‑versus‑column reciprocal comparisons. “#” indicate the FDA approved doses.

**Table S6. League table of phonophobia at 2h**

League table presenting pairwise relative effects across treatments (Risk Ratio with 95% CI), where each cell compares the treatment in the column against the treatment in the row. The upper‑right triangle shows column‑versus‑row estimates, and the lower‑left triangle shows the corresponding row‑versus‑column reciprocal comparisons. “#” indicate the FDA approved doses.

**Table S7. League table of Nausea at 2h**

League table presenting pairwise relative effects across treatments (Risk Ratio with 95% CI), where each cell compares the treatment in the column against the treatment in the row. The upper‑right triangle shows column‑versus‑row estimates, and the lower‑left triangle shows the corresponding row‑versus‑column reciprocal comparisons. “#” indicate the FDA approved doses.

**Figure S5. Sensitivity analysis of the outcomes using random effect model. “#” indicate the FDA approved doses. AAC: acetaminophen 250 mg/500 mg, aspirin 250 mg/500 mg, caffeine 65 mg/130 mg.**

**Table S8: Treatment-emergent adverse events (TEAEs)**

AAC: Acetaminophen, Aspirin, and Caffeine; Pbo: Placebo; Ibu: Ibuprofen; Ubro; Ubrogepant; Rimi: Rimegepant; Las: Lasmiditan, AEs: Adverse Event; TEAEs: Treatment emergent adverse events; TRAEs: Treatment related adverse events; URTI: Upper Respiratory Tract Infection; AST: Aspartate Aminotransferase and ALT: Alanine Aminotransferase. FDA approved doses include: AAC, Ibuprofen 200mg and 400mg, Lasmiditan 50mg,100mg and 200mg, Rimegepant 75mg, Ubrogepant 50mg and 100mg.
